# *Onychocamptus* Daday, 1903 from Thailand, with descriptions of two new species and two new records (Crustacea, Copepoda, Harpacticoida, Laophontidae)

**DOI:** 10.3897/zookeys.810.29253

**Published:** 2018-12-20

**Authors:** Chaichat Boonyanusith, Thanida Saetang, Koraon Wongkamheng, Supiyanit Maiphae

**Affiliations:** 1 School of Biology, Faculty of Science and Technology, Nakhon Ratchasima Rajabhat University, Nakhon Ratchasima, 30000, Thailand Nakhon Ratchasima Rajabhat University Nakhon Ratchasima Thailand; 2 Department of Zoology, Faculty of Science, Kasetsart University, Lad Yao, Chatuchak, Bangkok, 10900, Thailand Kasetsart University Bangkok Thailand

**Keywords:** Laophontidae, Southeast Asia, stygobiont

## Abstract

In this paper, two new species of *Onychocamptus* Daday, 1903 are described from Thailand: *Onychocamptussatunensis***sp. n.** and *Onychocamptustratensis***sp. n.** The following features mainly distinguish *O.satunensis***sp. n.** from known species: internal sausage-like and internal rounded structures on cephalothorax and one outer seta on the male P5 exopod that is as long as the supporting segment. In contrast, the cephalothorax of *O.tratensis***sp. n.** is smooth but has rounded integumental window-like structures, and the outer seta on the male P5 exopod is two times as long as the supporting segment. *Onychocamptusanomalus* shows the highest similarity with the two new species, but in contrast to both Thai species, it has only one seta on the exopod of the antenna. In addition, in the present study, two additional species, *O.bengalensis* and *O.vitiospinulosa*, are newly recorded in Thailand. Thus, the number of *Onychocamptus* species recorded in Thailand increases to five species. A key to all known species of this genus in the world is also proposed.

## Introduction

The genus *Onychocamptus* comprises eight species: (1) *Onychocamptusmohammed* (Blanchard & Richard, 1891), the type species, with *O.heteropus* Daday, 1903 considered a junior synonym (Zykoff 1904: 247, Lang 1948: 520, Huys 2009: 84); (2) *O.bengalensis* (Sewell, 1934); (3) *O.besnardi* Jakobi, 1954; (4) *O.vitiospinulosa* (Shen & Tai, 1963); (5) *O.anomalus* (Ranga Reddy, 1984); (6) *O.taifensis* (Kikuchi, Dai & Itô, 1993); (7) *O.krusensterni* (Schizas & Shirley, 1994), and (8) *O.fratrisaustralis* (Gómez, 2001). Most of the species are widely distributed in various types of inland waters, from fresh to saline (Lee and Chang 2005).

In Thailand, only one species, *O.mohammed*, had been reported (Apostolov 2007). However, from a more intensive study of harpacticoids in Thailand, including both intensive sampling and detailed study of the morphological characteristics, four more species of *Onychocamptus* have been found: *O.bengalensis* and *O.vitiospinulosa* are newly recorded in Thailand, and *O.satunensis* sp. n. and *O.tratensis* sp. n. are proposed as new species. This paper presents the joint results of two different research projects, a study on the diversity of cave-dwelling copepods in Satun province and a study on the diversity of copepods in important surface water bodies throughout Thailand. This study represents the first attempt to revise our knowledge on the diversity of this genus in Thailand.

## Materials and methods

**Figure 1.**
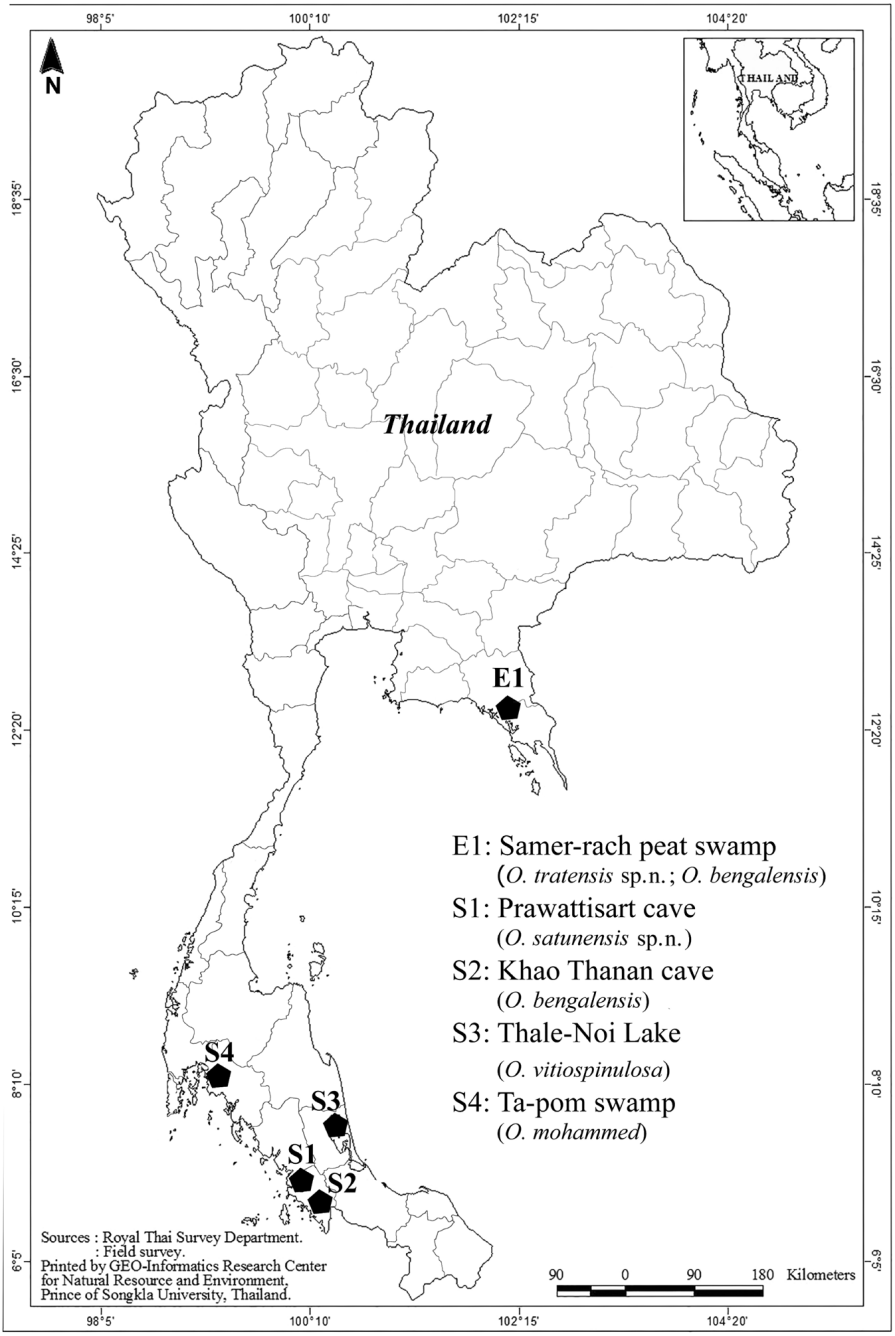
Sampling sites.

Plankton samples were collected from the Samer-rach peat swamp (Trat Province) in eastern Thailand, and from the Prawattisart and Khao Thanan caves (Satun Province), Thale-Noi Lake (Pattalung Province), and Ta-pom swamp (Krabi Province) in southern Thailand (Fig. 1). A plankton net with a 60-µm mesh was used for collecting the samples, which were immediately preserved in 70% ethanol. The copepods were sorted using an Olympus SZ-40 stereo microscope, and identified specimens of *Onychocamptus* were dissected and mounted on a slide, with glycerine as the mounting medium, and sealed with nail varnish. Drawings were made from both complete and dissected specimens using a camera lucida attached to an Olympus CH-2 compound microscope. For scanning electron microscopy, the samples were dehydrated in a series of increasing ethanol concentrations: 60%, 80%, 90%, 95%, 96%, and 100%. The samples were dehydrated twice in each concentration, for 15 min each time. Then, the specimens were subjected to the critical point drying process, mounted on stubs, coated with gold, and examined with a scanning electron microscope (Quanta 450 FEI). The descriptive terminology proposed by Huys et al. (1996) and the armature formula P1-P4 proposed by Sewell (1949) (cited by Huys and Boxshall 1991) were adopted. P1–P6, swimming legs 1–6; enp-1 (2, 3), proximal (middle, distal) segment of the endopod; and exp-1 (2, 3), proximal (middle, distal) segment of the exopod. Holotype, allotype, and paratypes were deposited in the reference collection of the Princess Maha Chakri Sirindhorn National History Museum, Prince of Songkla University, Songkhla, Thailand (**PSUNHM**). Voucher specimens were deposited in the collections of the first author (CB) and in the crustacean reference collection of the Zoological Museum, Kasetsart University (**ZMKU_CP**).

## Taxonomy

### Order Harpacticoida Sars, 1903

#### Family Laophontidae T. Scott, 1904

##### Genus *Onychocamptus* Daday, 1903

###### Onychocamptus satunensis sp. n.

Taxon classificationAnimaliaHarpacticoidaLaophontidae

http://zoobank.org/F38757B6-A85E-4F7C-B4AC-520821E6C650

Figs 2 , 3 , 4 , 5 , 6 , 7 , 8 , 23A

####### Material examined.

**Holotype.** Adult female, dissected and mounted onto one slide, collected from type locality on 30 July 2015 (PSUZC-PK2002-01). **Allotype.** One adult male, dissected and mounted onto one slide, (PSUZC-PK2002-02), all collected from type locality on 20 January 2016. **Paratypes**. One undissected adult ovigerous female paratype mounted onto one slide (PSUZC-PK2002-03), and one undissected adult male paratype, mounted onto one slide (PSUZC-PK2002-04), coll. C Boonyanusith and K Wongkamheng; type locality on 30 July 2015 and 20 January 2016, respectively.

####### Additional material.

One adult ovigerous female and one adult male, both collected from type locality on 2 April 2015 and stored in 70% ethanol, deposited in the collection of the first author (CB).

####### Type locality.

Prawattisart cave, Muang district, Satun Province, southern Thailand, 6°42'55.82"N, 100°05'19.17"E. The cave is in an isolated, wedge-shaped, limestone hill. It is surrounded by irrigation canal and paddy fields, but there are no connections between water in and outside cave. Beyond entrance of the cave there is a long horizontal gallery, with approximately 5–10 m high and approximately 2–3 m wide. At some place there are two openings, which are large enough for entry, indicating a complex tunnel system. The collecting point is a part of a large water body inside the cave, fulfilled by approximately 10–50 cm depth of water. Water depth varies according to season, but it has never dried out. Water was turbid and flowed slowly. Water temperature was 25.5 °C, pH 7.8, conductivity 360 µS cm^-1^, dissolved oxygen 4.9 mgL^-1^.

####### Etymology.

The specific name *satunensis* is derived from the name of Satun Province, where the species was collected. The name is a noun in the genitive singular, masculine.

####### Differential diagnosis.

Laophontidae. Body gradually tapering posteriorly. Cephalothorax with internal sausage-like structure, and internal rounded structures. Posterior margin of cephalothorax and body somites (except penultimate and anal somite) with sensillum-bearing tubercles. Second and third urosomite partly fused ventrally in female, representing genital double-somite. Caudal rami approximately 2.5 times as long as wide, with one transverse inner row of spinules near insertion of dorsal seta. Caudal seta IV and V fused. Allobasis of antenna without abexopodal seta. Endopodal lobe of P5 with three, and exopod with four pinnate setae. Male P3 enp-2 with apophysis on outer distal corner, reaching tip of enp-3. Exopod of male P5 with three setae, outer seta as long as supporting segment. Male P6 reduced, with outer seta and inner bipinnate seta apically; inner seta approximately twice as long as outer one.

####### Description of adult female.

**Figure 2.**
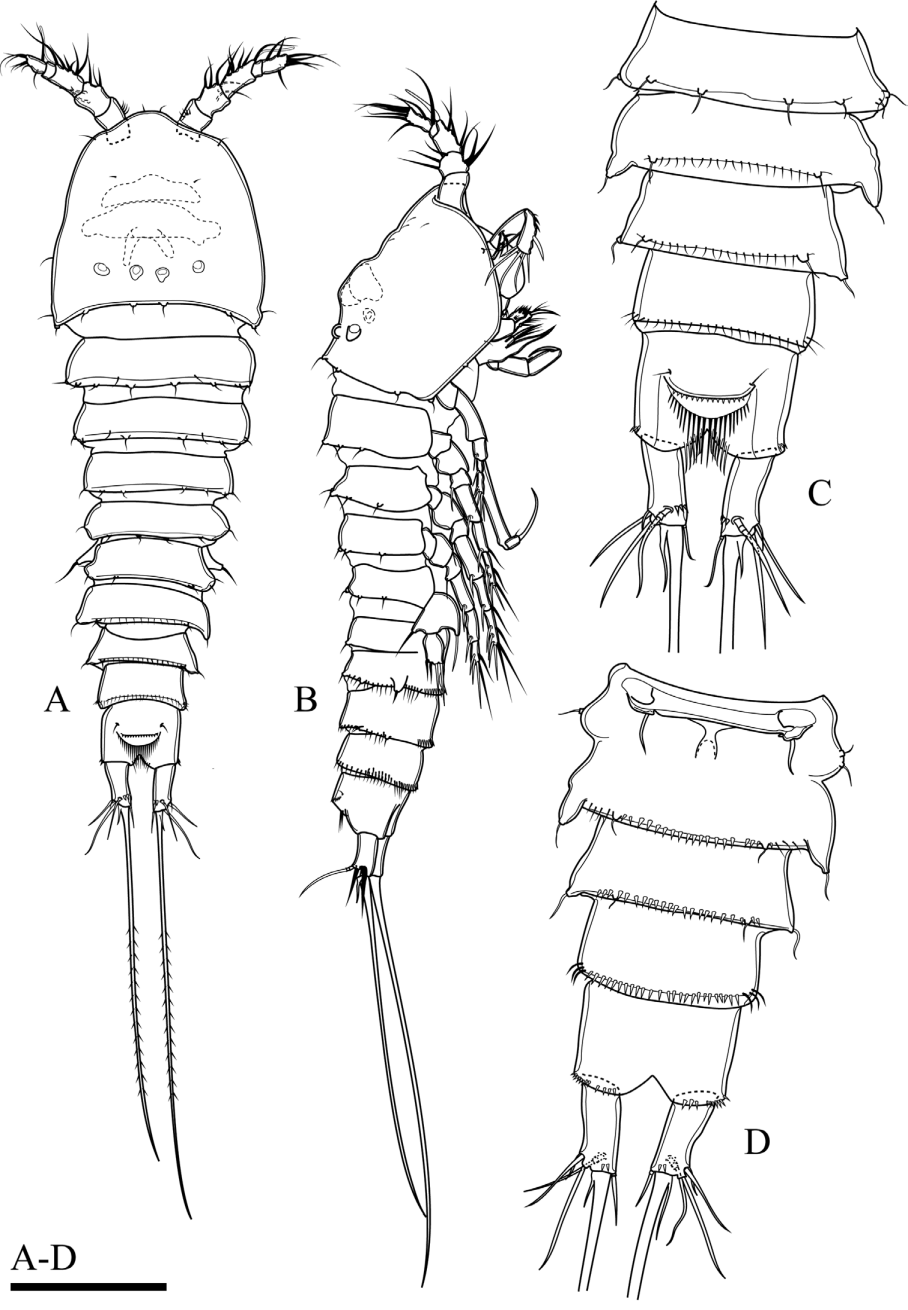
*Onychocamptussatunensis* sp. n., female holotype. **A** habitus, dorsal view **B** habitus, lateral view **C** urosome, dorsal view **D** urosome, ventral view. Scale bar: 0.1 mm (**A, B**), 0.05 mm (**C, D**).

**Figure 3.**
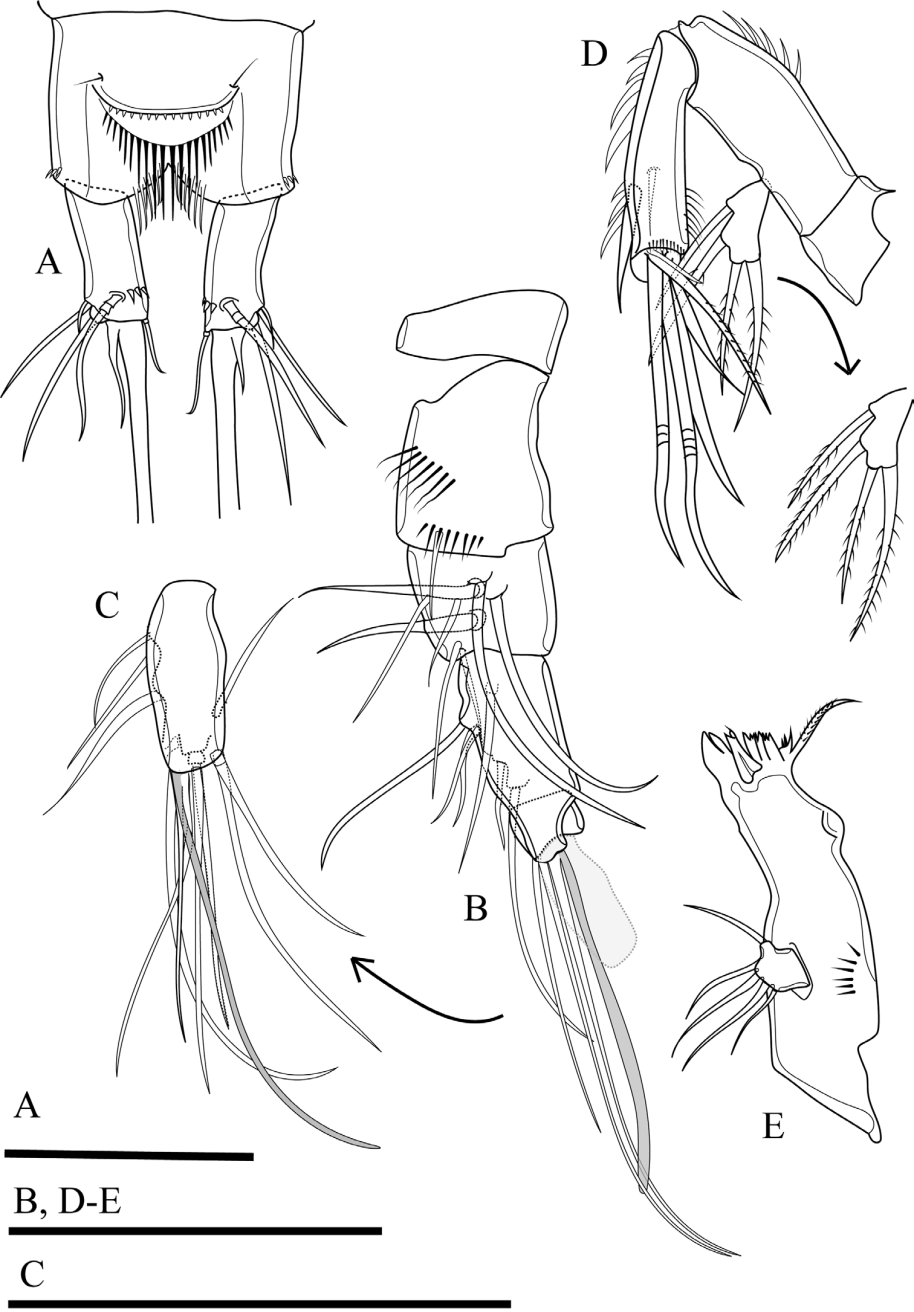
*Onychocamptussatunensis* sp. n., female holotype. **A** anal somite, dorsal view **B** antennule **C** ultimate segment of antennule **D** antenna **E** mandible. Scale bars: 0.05 mm.

Body (Fig. 2A). Total body length, measured from tip of rostrum to posterior margin of caudal rami, 401–445 µm (mean 419 µm, n = 3; 445 µm in holotype); preserved specimen colourless. Body covered with setules (Fig. 8E, F), cylindrical; gradually tapering posteriorly, with maximum width at posterior part of cephalothorax. Prosome approximately 1.3 times as long as urosome (including caudal rami) (Fig. 2A). Rostrum small, completely fused to cephalothorax. Cephalothorax as long as wide, approximately 0.5 times the length of prosome, just under integument with anterior internal structure comprising three parts; each of which sausage-like, and with internal rounded structures near distal margin (Figs 2A, B, 8A, B). Cephalothorax and all free thoracic somites with sensillum-bearing tubercles along posterior margin (Fig. 2A, B). Second and third urosomite fused ventrally (Fig. 2D), distinct dorsally and laterally (Fig. 2A–C), original division between sixth thoracic somite and first abdominal somite, with dorsal sensillum-bearing tubercles (Fig. 2C). Genital field ribbon-shaped, with seta representing P6 at outer distal corner (Fig. 2D). The remnant of first abdominal somite (posterior half of genital double-somite) with lateral sensillum-bearing tubercles (Fig. 2C, D). Posterior margin of genital double-somite and fourth urosomites with outer sensillum-bearing tubercles; posterior half of genital double-somite and fourth urosomite with posterior setules dorsally (Fig. 2C) and small spinules ventrally (Fig. 2D) between sensillum-bearing tubercles. Penultimate urosomite with posterior setules dorsally and laterally, with one posterior row of spinules ventrally. Anal somite as long as wide, with arch row of long spinules posterior to anal operculum (Figs 2A, C, 3A, 8D), with ventrolateral row of minute spinules near insertion of caudal rami (Fig. 2D). Anal operculum poorly developed, with minute spinules along posterior margin (Figs 2C, 3A, 8D).

Caudal rami (Figs 2C, D, 3A). Slightly convergent, 2.5 times as long as wide, with one transverse row of inner spinules near insertion of caudal seta (VII) (Figs 2C, 3A). Anterolateral accessory seta (I) minute, close to anterolateral seta (II), both subapical. Posterolateral seta (III) inserted on minute pedestal. Outer terminal seta (IV) slender, fused at base with inner terminal seta (V), the latter longest, without fracture plane, approximately 0.6 times as long as body length. Inner accessory seta (VI) slender. Dorsal seta (VII) tri-articulate, inserted at quarter of rami. Length ratio of caudal setae to ramus length, from seta I to seta VII of the holotype: 0.4 : 0.8 : 1.3 : 0.7 : 9.7 : 0.7 : 1.1.

The ovigerous female bears one oval egg sac with eight eggs, located ventrally between pair of fifth legs.

Antennule (Fig. 3B, C). Short, 5-segmented. First segment with medial and distal rows of spinules. Armature formula: I-[1], II-[8], III-[7+(1+aesthetasc)], IV-[1], V-[9+acrothek]. Aesthetasc on third segment robust, fused basally to one seta. Apical acrothek on fifth segment slender, consisting of one aesthetasc fused basally to two slender smooth seta.

Antenna (Fig. 3D). Comprising coxa, allobasis, and 1-segmented endopod and exopod. Coxa without ornamentation. Allobasis with one row of inner spinules, with 1-segmented exopod; the latter with two apical and two lateral bipinnate setae. Free endopodal segment with one strong, sharp spine, and one seta at distal third of segment; the former accompanied by several strong, short spinules; distal end with six elements: one minute seta, one slender, bipinnate seta, two geniculate setae, and two strong, smooth spines.

Mandible (Fig. 3E). Gnathobase with strong, chitinised teeth and lateral pinnate seta. Mandibular palp short, 1-segmented, with five slender setae sub-equal in length.

**Figure 4.**
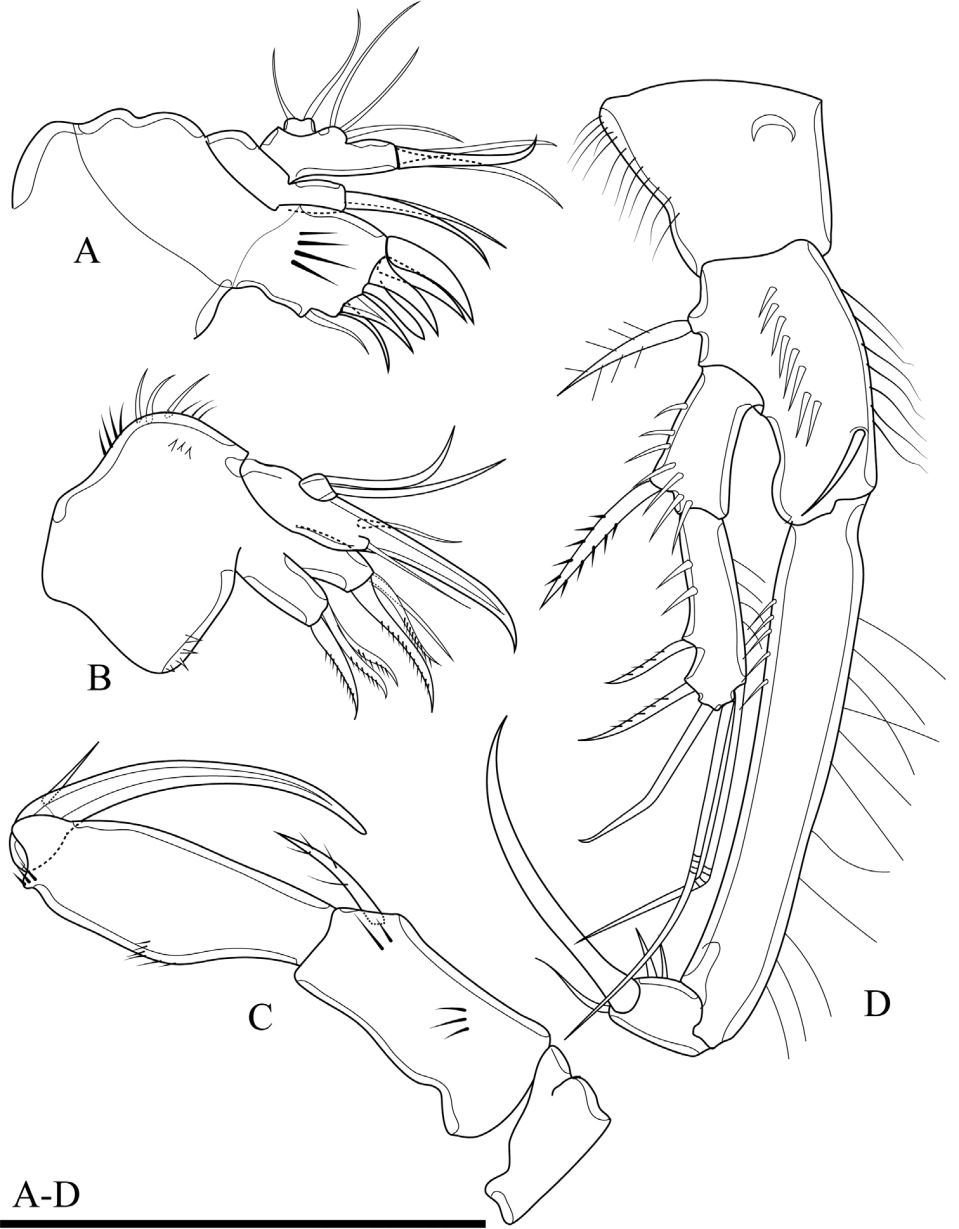
*Onychocamptussatunensis* sp. n., female holotype. **A** maxillule **B** maxilla **C** maxilliped **D** P1. Scale bar: 0.05 mm.

Maxillule (Fig. 4A). Composed of robust precoxa, coxa, basis, endopod fused to basis, and 1-segmented free exopod. Precoxal arthrite with six strong apical spines and one slender lateral seta. Coxa with cylindrical endite bearing two smooth setae, one of which robust. Basis with cylindrical endite bearing three setae, one of which robust. Endopod incorporated to basis, with three setae. Exopod free, 1-segmented, with two sub-equal apical setae.

Maxilla (Fig. 4B). Composed of syncoxa, allobasis, and 1-segmented endopod. Syncoxa with two endites; proximal endite with three apical pinnate setae, distal endite with two pinnate and one slender apical seta, outer margin with spinules. Allobasis with apical drawn out into claw, with one anterior and one posterior seta. Endopod 1-segmented, with two smooth apical setae.

Maxilliped (Fig. 4C). Subchelate, 3-segmented, comprising syncoxa, basis, and endopod. Syncoxa with one pinnate seta at inner distal corner. Basis with two transverse rows of outer spinules, one of which near base of endopod. Endopod drawn out into strong claw, with one minute seta near its base.

P1 (Fig. 4D). Coxa with longitudinal row of outer setules. Basis with one outer bipinnate spine and one inner spine near insertion of endopod, with longitudinal row of anterior spinules medially, with long setules along inner margin. Both rami 2-segmented. Exopod reaching proximal third of enp-1; exp-1 with one bipinnate outer spine, with one row of outer spinules; exp-2 with three outer spines, two apical geniculate setae, with outer spinules and inner setules. Enp-1 approximately seven times as long as wide, with outer spinules and inner setules; enp-2 with one median strong outwardly curved claw-like smooth spine and one slender inner seta, with few outer spinules.

**Figure 5.**
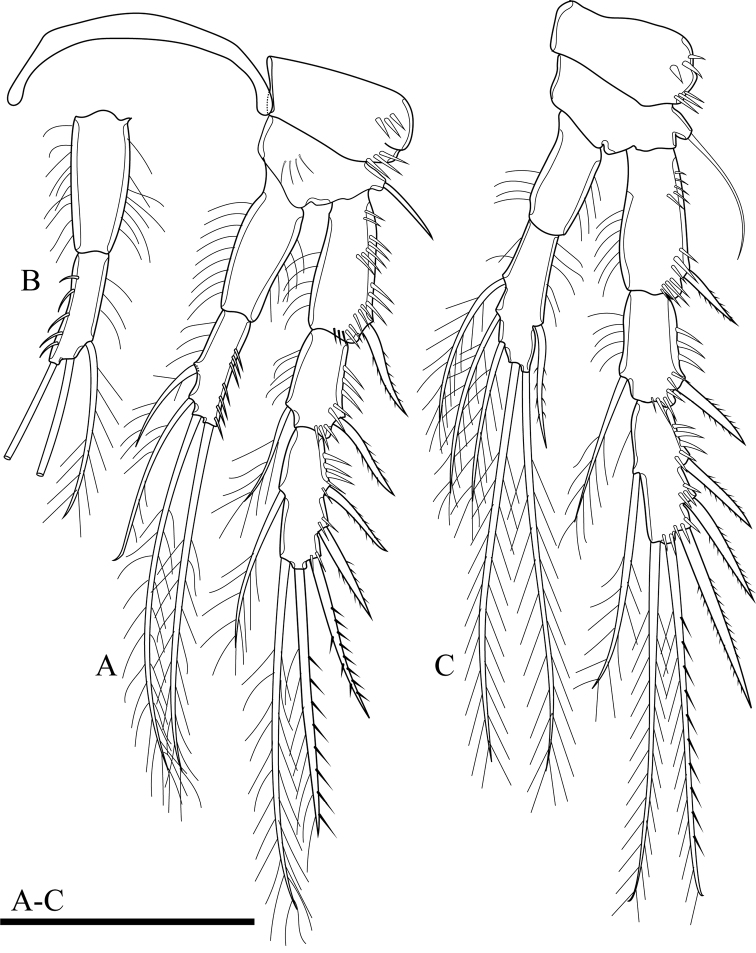
*Onychocamptussatunensis* sp. n., female holotype. **A** P2 **B** right P2 endopod **C** P3. Scale bar: 0.05 mm.

P2 (Fig. 5A, B). Coxa with oblique row of spinules on anterior surface near outer margin, with row of spinules at distal outer corner. Basis with outer spine. Rami with 3-segmented exopod and 2-segmented endopod; endopod reaching tip of exp-2. Exp-1 with one outer bipinnate spine; exp-2 with one outer bipinnate spine and one inner plumose seta; exp-3 with three outer bipinnate spines, two apical elements (of which outer one spiniform seta with outer spinules and inner setules, inner element one plumose seta), and one inner plumose seta. All segments of exopod with row of outer spinules and inner setules. Enp-1 without armature, enp-2 with two apical and two inner plumose setae. All segments of endopod with row of outer spinules and long inner setules.

P3 (Fig. 5C). Coxa, basis, and segmentation of rami as in P2. Outer element of basis one long seta. Endopod reaching tip of exp-2. Exp-1 with one outer bipinnate spine; exp-2 with one outer bipinnate spine and one inner plumose seta; exp-3 with three outer bipinnate spines, two apical elements (of which outer one spiniform seta with outer spinules and inner setules, inner element one plumose seta), and one inner plumose seta. Ornamentation of exopod as in P2. Enp-1 without armature; enp-2 with one outer bipinnate seta, two apical, and three inner plumose setae. Outer and inner margins of segments of endopod with setules.

**Figure 6.**
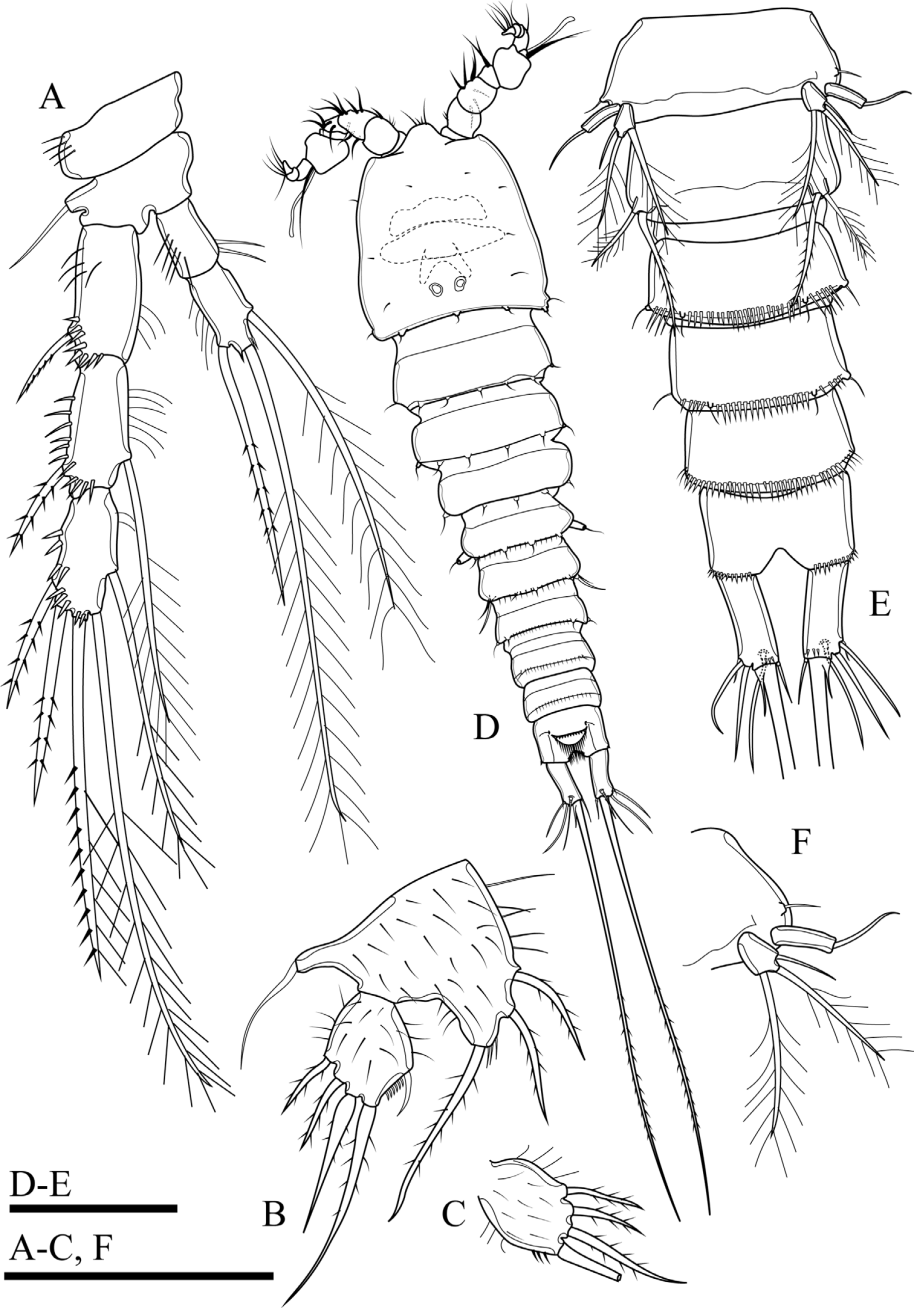
*Onychocamptussatunensis* sp. n., female holotype. **A** P4 **B** right P5 **C** left P5 exopod **D***Onychocamptussatunensis* sp. n., male allotype. habitus, dorsal view **E** urosome, ventral view **F** left P5. Scale bars: 0.05 mm.

P4 (Fig. 6A). Coxa with row of outer long spinules. Basis with one slender outer seta. Rami with 3-segmented exopod and 2-segmented endopod; endopod reaching tip of exp-1. Exp-1 with one outer bipinnate spine; exp-2 with one outer bipinnate spine and one inner plumose seta; exp-3 with two outer bipinnate spines, two apical elements (of which outer one spiniform seta with outer spinules and inner setules, inner element one plumose seta), and one inner plumose seta. Ornamentation of exopod as in P2 and P3. Enp-1 without armature, with long inner subdistal spinules and smaller outer spinules; enp-2 with one outer bipinnate seta, one apical plumose seta, and one inner plumose seta.

Armature formula of P1−P4 as in Table 1.

**Table 1.** Armature formula of P1–P4 of *Onychocamptussatunensis* sp. n. and *Onychocamptustratensis* sp. n.

Swimming legs		Basis	Exopod	Endopod
P1	female	I-I	I-0; III,2,0	0-0; 0,I1,0
	male	I-I	I-0; III,2,0	0-0; 0,I1,0
P2	female	I-0	I-0; I-1; III,2,1	0-0; 0,2,2
	male	I-0	I-0; I-1; III,2,1	0-0; 0,2,2
P3	female	1-0	I-0; I-1; III,2,1	0-0; 1,2,3
	male	1-0	I-0; I-1; III,2,1	0-0; 0-1; 0,2,2
P4	female	1-0	I-0; I-1; II,2,1	0-0; 1,1,1
	male	1-0	I-0; I-1; II,2,1	0-0; 1,1,1

P5 (Figs 6B, C, 23A). Baseoendopod and exopod separated, with setules as on figures. Baseoendopod with basal seta and three pinnate setae on endopodal lobe, with spinules at base of distalmost seta. Exopod with four pinnate setae; innermost one longest.

P6 (Fig. 2D). Reduced to minute prominence at outer distal corner of genital field, with one short, slender seta.

####### Description of adult male.

Body (Fig. 6D). Total body length, measured from tip of rostrum to posterior margin of caudal rami, 352−410 µm (mean 374 µm, n = 3; 410 µm in allotype); preserved specimen colourless. Prosome approximately 1.3 times as long as urosome (Fig. 6D). Cephalothorax as long as wide, 0.5 times the length of prosome, internal sausage-like structure as in female, with two internal rounded structures. All free thoracic somites with sensillum-bearing tubercles along dorsal posterior margin, but fifth thoracic somite (first urosomite) with additional row of posterior setules dorsally (Fig. 6D). Second and third urosomite completely separated. Second urosomite with dorsal sensillum-bearing tubercles along posterior margin (Fig. 6D). Fourth urosomite without lateral protuberances, with lateral sensillum-bearing tubercles, with one posterior row of dorsal setules and ventral spinules. Ornamentation on next three urosomites as in female (Fig. 6E). Anal somite and anal operculum as in female.

Caudal rami as in female (Fig. 6D, E).

**Figure 7.**
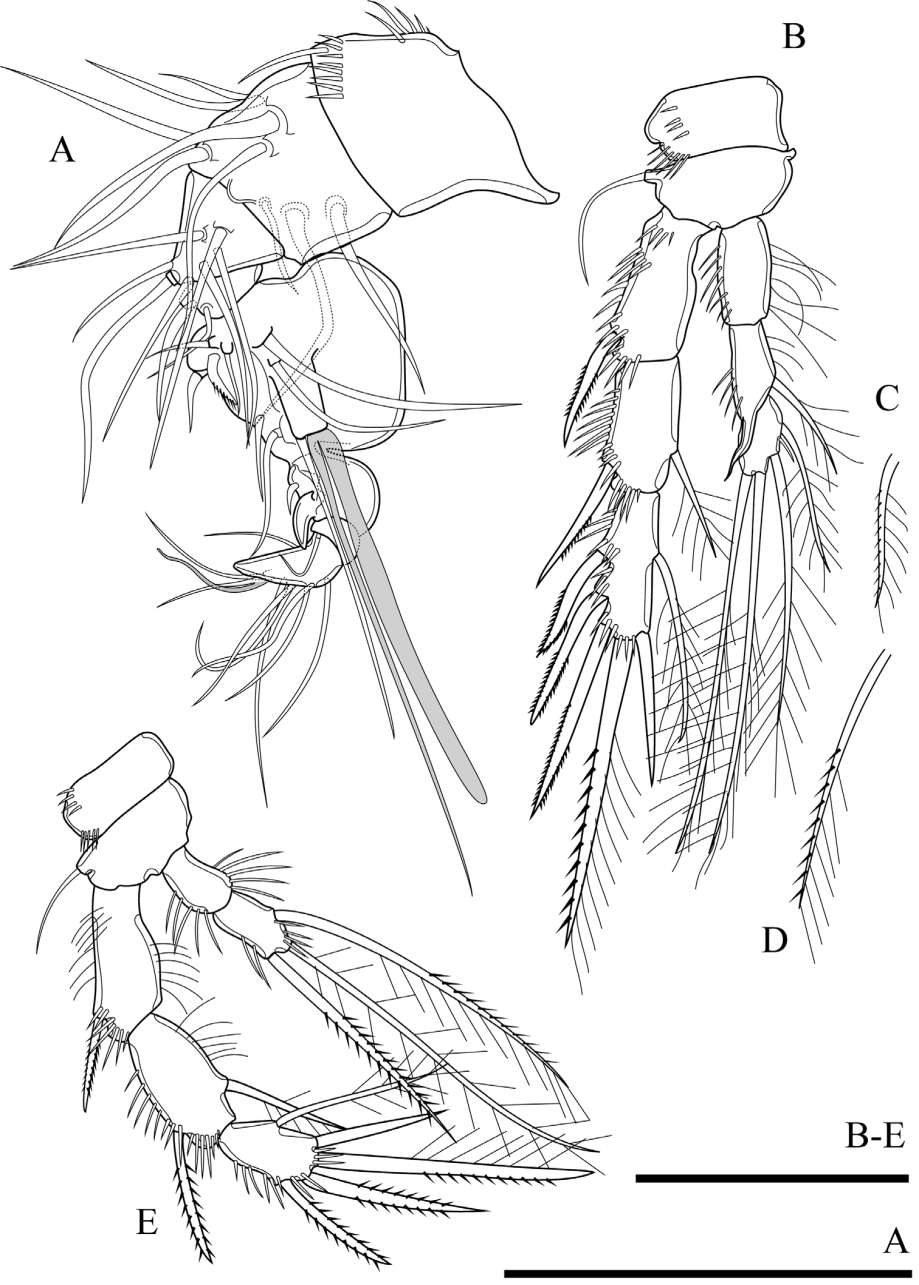
*Onychocamptussatunensis* sp. n., male allotype. **A** antennule **B** right P3 **C** proximal inner seta on left P3 enp-3 **D** distal inner seta on left P3 enp-3 **E** P4. Scale bars: 0.05 mm.

Antennule (Fig. 7A). 8-segmented, geniculate, with three segment distal to geniculation. First segment with proximal and subdistal outer spinules. Armature formula I-[1], II-[9], III-[7], IV-[2], V-[9+(1+aesthetasc)], VI-[1], VII-[1], VIII-[7+acrothek)]. Aesthetasc on fifth segment robust, fused basally to one seta. One pinnate seta on fifth segment. Apical acrothek on eighth segment small, consisting of one aesthetasc fused basally to two slender smooth seta.

Rostrum, antenna, mouthparts and P1, P2 (not shown) as in female.

P3 (Fig. 7B). General shape as in female but both rami 3-segmented. Endopod reaching tip of exp-2. Exp-3 with three outer bipinnate spines, two apical elements (of which outer one spiniform robust seta with outer spinules and inner setules, inner element one smooth spiniform seta), and one inner plumose seta. Enp-2 with inner seta and outer apophysis on outer distal corner reaching tip of enp-3, with long outer spinules and few inner setules; enp-3 with two inner and two apical plumose setae.

P4 (Fig. 7E). General shape as in female but exp-3 relatively shorter and with stronger elements. Exp-3 with two outer bipinnate spines, two apical elements (of which outer one spiniform robust seta with outer spinules and inner setules, inner element one spiniform seta), and one inner plumose seta.

Armature formula of P1–P4 as in Table 1.

P5 (Fig. 6E, F). With outer basal seta arising from long setophore; without endopodal lobe. Exopod with three setae, outermost slender and slightly longer than segment, approximately 1/3 times as long as the middle seta.

P6 (Fig. 6E). Reduced to one minute rectangular protuberance, with outer plumose seta and inner bipinnate seta; inner seta approximately twice as long as outer one and reaching posterior margin of next urosomite.

####### Variability.

The right P2 enp-2 lacks the proximalmost inner seta (Fig. 5B), and one additional small inner seta on the P5 exopod was observed (Fig. 6B) in the holotype. The P3 enp-3 of the allotype possesses two inner setae. Also, on the right ramus the setae possess outer and inner setules (Fig. 7B), but those of the left ramus possess inner spinules (Fig. 7C, D).

**Figure 8.**
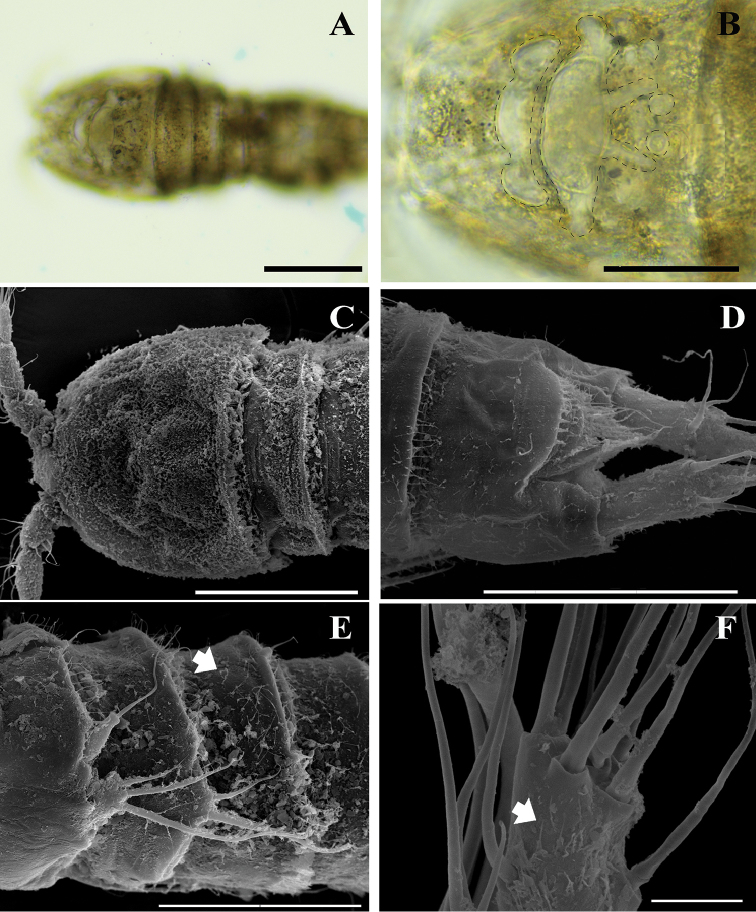
*Onychocamptussatunensis* sp. n., Digital photographs (**A, B**) and SEM photographs. **A–C** cephalothorax (dotted line on figure B indicates margin of internal structures) **D** anal somite and caudal rami **E** male urosome, lateral view, arrow indicates setules **F** tip of female antennule, arrow indicates setules. Scale bars: 0.1 mm (**A, C**), 0.05 mm (**B, D**), 0.005 mm (**E**), 0.02 mm (**F**).

*Onychocamptussatunensis* sp. n. is the only species of *Onychocamptus* with internal sausage−like structure on cephalothorax (Figs 2A, B, 8A–C), differing from other members. *O.satunensis* sp. n. is most similar to *O.tratensis* sp. n., both species sharing the following remarkably characters: 1) absence of abexpodal seta on allobasis, 2) presence of 4 setae on exopod of antenna, 3) presence of 2 outer spines on P4 exp-3, 4) presence of 4 setae on exopod of P5 of the female, and 5) presence of 3 setae on exopod of P5 of the male. The southern Thai species (*O.satunensis* sp. n.), however, can be distinguished from the eastern one (*O.tratensis* sp. n.) by the presence of internal sausage−like structure, the presence of internal rounded structures, and the relative length of outer seta on P3 enp-2. Comparative study between two Thai new species and their congeners is provided in Table 3.

**Table 3.** Comparison of characters of female and male of genus *Onychocamptus*.

	* O. anomalus *	* O. bengalensis *	* O. besnardi *	* O. fratrisaustralis *	* O. krusensterni *	* O. mohammed *	* O. taifensis *	* O. vitiospinulosa *	*O.satunensis* sp. n.	*O.tratensis* sp. n.
Ornamentation on cephalothorax	smooth	smooth	smooth	smooth	smooth	smooth	smooth	smooth	**internal sausage-like structure, and internal rounded structures**	**1 middle and 2 lateral rounded integumental window-like structures**
Exp of antenna (setae)	1, reduced	4, well developed	4, well developed	4, well developed	4, well developed	4, well developed	4, well developed	4, well developed	4, well developed	4, well developed
Lateral seta on exp of antenna	complete absent	pinnate spinulose seta	NA	pinnate spinulose seta	pinnate spinulose, seta	NA	bare, slender, short seta	bare, slender, short seta	pinnate spinulose, seta	pinnate spinulose seta
Abexopodal seta of antenna	absent	present	present	present	present	present	absent	present	absent	absent
P2 enp-2 (inner setae)	2	2	2	2	2	2	1	2	2	2
P4 exp-3 (outer spines)	2	3	2	2	2	3	3	3	2	2
P4 exp-3 (inner seta)	present	present	absent	present	present	present	present	present	present	present
Female P5 exp and beseoendopod	separated	fused	separated*	separated	separated	separated	separated	separated	separated	separated
Setae on female P5 exp:enp	4:3	3:3	3:3	3:3	3:3	3:3	3:3	3:2	4:3	4:3
Setae on male P5 exp	3	2	2	NA	2	2	2	2	3	3
Male P5, outer seta of exp	A	C	NA	NA	C	C	B	B	**D**	**E**
CR (L:W ratio)	2.7	3.0–6.0	2	2.2–3.0	2.0–3.0	2.4–3	1.8–2.0	1.8–2.2	2.5	2.2
Seta of male P6	a	d	NA	NA	c	c	e	b	a	a
Segment of male antennule	6	6–7	?	NA	6–7	7	7	7–8	8	8
Distribution	India	India, Japan, Korea, Australia	Brazil, Micronesia, NW Mexico	NW Mexico	Alaska	Cosmo-politan	China and Korea	Korea	Thailand	Thailand
Ecology	Lake	Estuary	With marine algae	Estuary	Lagoon, 1 m	Cave, FW	Lake	Inland water	Cave	Peatswamp

Note: ? = doubtful. **NA** = not available. * = separated, noted in written description but fused in figure (Jakobi 1954: 197-198, 211, pl.VI, Figs 1–15). A = long; outer seta 4 times as long as supporting segment; outer and middle setae sub-equal; B = seta not reaching beyond second urosomite, sub-equal in length; C = reaching beyond second urosomite, sub-equal in length; D = short, outer seta as long as supporting segment, 1/3 as long as the middle seta; E = outer seta 2 times as long as supporting segment, 1/2 as long as the middle seta. **Male P6**, length of seta of male P6, a = inner seta long, reaching beyond distal margin of Ab1; outer seta 1/2 times as long as inner seta length; b = inner seta long extending beyond third urosomite; outer seta 1/4-1/2 as long as inner seta; c = inner seta long reaching beyond third urosomite; outer seta 3/4 times as long as inner seta; d = inner seta long, reaching beyond third urosomite; outer and inner seta sub-equal in length; e = inner and outer seta relatively.

####### Distribution.

This species is known from the type locality only.

###### Onychocamptus tratensis sp. n.

Taxon classificationAnimaliaHarpacticoidaLaophontidae

http://zoobank.org/3C3184B8-A779-436B-87BA-B009F324F54E

Figs 9 , 10 , 11 , 12 , 13 , 14 , 15 , 22A , 23B

####### Material examined.

**Holotype.** Adult female, dissected and mounted onto two slides (PSUZC-PK2003-01–PSUZC-PK2003-02). **Allotype.** One adult male, dissected and mounted onto two slides (PSUZC-PK2003-03–PSUZC-PK2003-04). **Paratypes.** One undissected adult female, mounted onto one slide, (PSUZC-PK2003-05). One undissected adult male, mounted onto one slide, (PSUZC-PK2003-06). One adult female, dissected and mounted onto two slides, (PSUZC-PK2003-07–PSUZC-PK2003-08).

All specimens were collected by S Maiphae and T Saetang from type locality on 9 January 2017.

####### Additional material.

Ten females and five males, all collected from type locality on 9 January 2017 and stored in 70% ethanol, deposited in crustacean reference collection, Zoological Museum, Kasetsart University (ZMKU_CP).

####### Type locality.

Samer-rach peat swamp, Trat Province, eastern Thailand, 12°28'04.0"N, 102°21'20.6"E. Water temperature ranged between 28.83 °C, pH of 6.23, salinity 6.91 ppt, total dissolved solids 7.9 mg L^-1^, and dissolved oxygen 4.41 mg L^-1^.

####### Etymology.

The specific name *tratensis* is derived from the name of Trat Province, where the species was collected. The name is a noun in the genitive singular, masculine.

####### Differential diagnosis.

Laophontidae. Body gradually tapering posteriorly. One middle and two lateral rounded integumental window-like structures on cephalothorax. Second and third urosomite fused ventrally in female forming genital double-somite. Caudal rami cylindrical, both sides parallel, approximately 2.5 twice as long as wide, with one longitudinal row of minute spinules on inner margin near insertion of dorsal seta and horizontal row of minute spinules near insertion of inner terminal seta. Outer terminal seta (seta IV) fused at base with inner terminal seta. Allobasis of antenna without abexopodal seta. Endopodal lobe of P5 with two bipinnate and one plumose setae on inner margin and exopod of P5 with four plumose setae. Enp-2 of male P3 with apophysis on outer distal corner; apophysis reaching the tip of enp-3. Exopod of male P5 with three bipinnate setae, outer seta two times as long as supporting segment. P6 of the male reduced, with outer seta and inner bipinnate seta apically; inner seta approximately twice as long as outer one.

####### Description of adult female.

**Figure 9.**
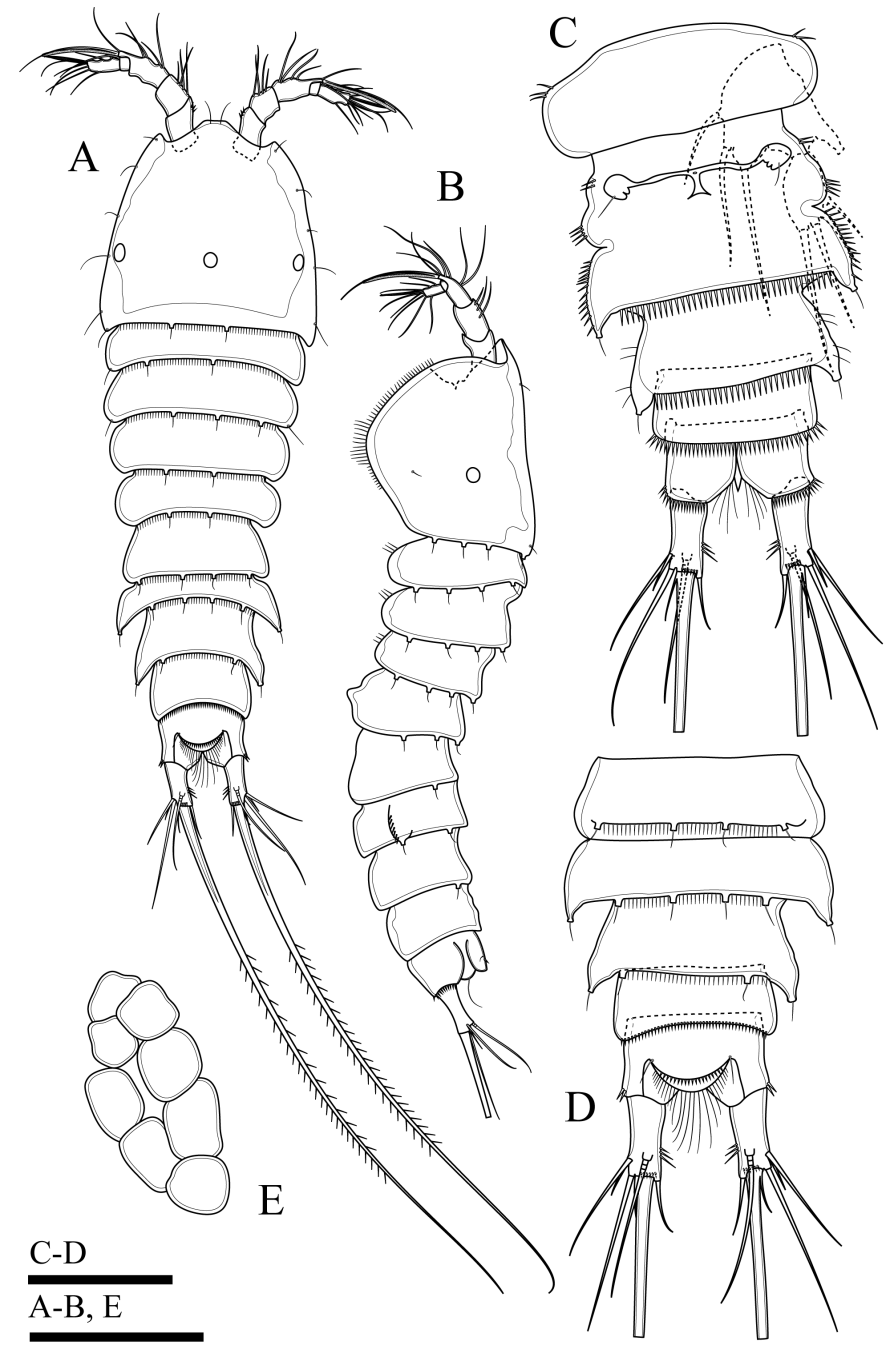
*Onychocamptustratensis* sp. n., female holotype. **A** habitus, dorsal view **B** habitus, lateral view **C** urosome, ventral view **D** urosome, dorsal view **E** egg sac. Scale bars: 0.1 mm (**A, B, E**), 0.05 mm (**C, D**).

Body. Total body length, measured from tip of rostrum to posterior margin of caudal rami, 360–450 µm (mean 400 µm, n = 14; 420 µm in holotype); preserved specimen colourless. Body covered entirely with setules, cylindrical; gradually tapering posteriorly, with maximum width at posterior part of cephalothorax. Prosome 1.3 times as long as urosome (including caudal rami) (Fig. 9A). Rostrum small, completely fused to cephalothorax. Cephalothorax as long as wide, approximately 0.5 times the length of prosome length, with one middle and two lateral rounded integumental window-like structures on cephalothorax. Cephalothorax and all free thoracic somites with sensillum-bearing tubercles along posterior margin. Second and third urosomite fused ventrally (Fig. 9C, D), distinct dorsally. Genital field ribbon-shaped, with seta representing P6 at outer distal corner (Fig. 9C). The remnant of first abdominal somite (posterior half of genital double-somite) with lateral sensillum-bearing tubercles (Fig. 9C, D). Posterior margin of genital double-somite and fourth urosomites with outer sensillum-bearing tubercles; posterior half of genital double-somite and fourth urosomite with posterior setules dorsally (Fig. 9D) and small spinules ventrally (Fig. 9C) between sensillum-bearing tubercles. The penultimate urosomite with posterior setules dorsally and laterally, with one posterior row of spinules ventrally. Anal somite approximately 0.6 times longer than wide, with arch row of long spinules posterior to anal operculum (Fig. 9D), with ventrolateral row of minute spinules near insertion of caudal rami (Fig. 9C). Anal operculum poorly developed, with minute spinules along posterior margin (Fig. 9D).

Caudal rami (Fig. 9C, D). Cylindrical, both sides parallel, 2.5 times as long as wide, with one longitudinal row of minute inner spinules near insertion of caudal seta (VII) and horizontal row of minute spinules near insertion of inner terminal seta (V). Anterolateral accessory seta (I) minute, inserted, close to anterolateral seta (II), both subapical. Posterolateral seta (III) inserted on minute pedestal. Outer terminal seta (IV) slender, fused at base with inner terminal seta (V), the letter longest, without fracture plane, approximately 0.9 times as long as body length. Inner accessory seta (VI) slender. Dorsal seta (VII) tri-articulate, inserted at quarter of rami. Length ratio of caudal setae to ramus length, from seta I to seta VII of holotype: 0.5 : 1.7 : 2.5 : 0.7 : 13.3 : 0.7 : 2.0.

Egg sac (Fig. 9E). Ovigerous female with two egg sacs ventrally between pair of P5, each with eight eggs.

**Figure 10.**
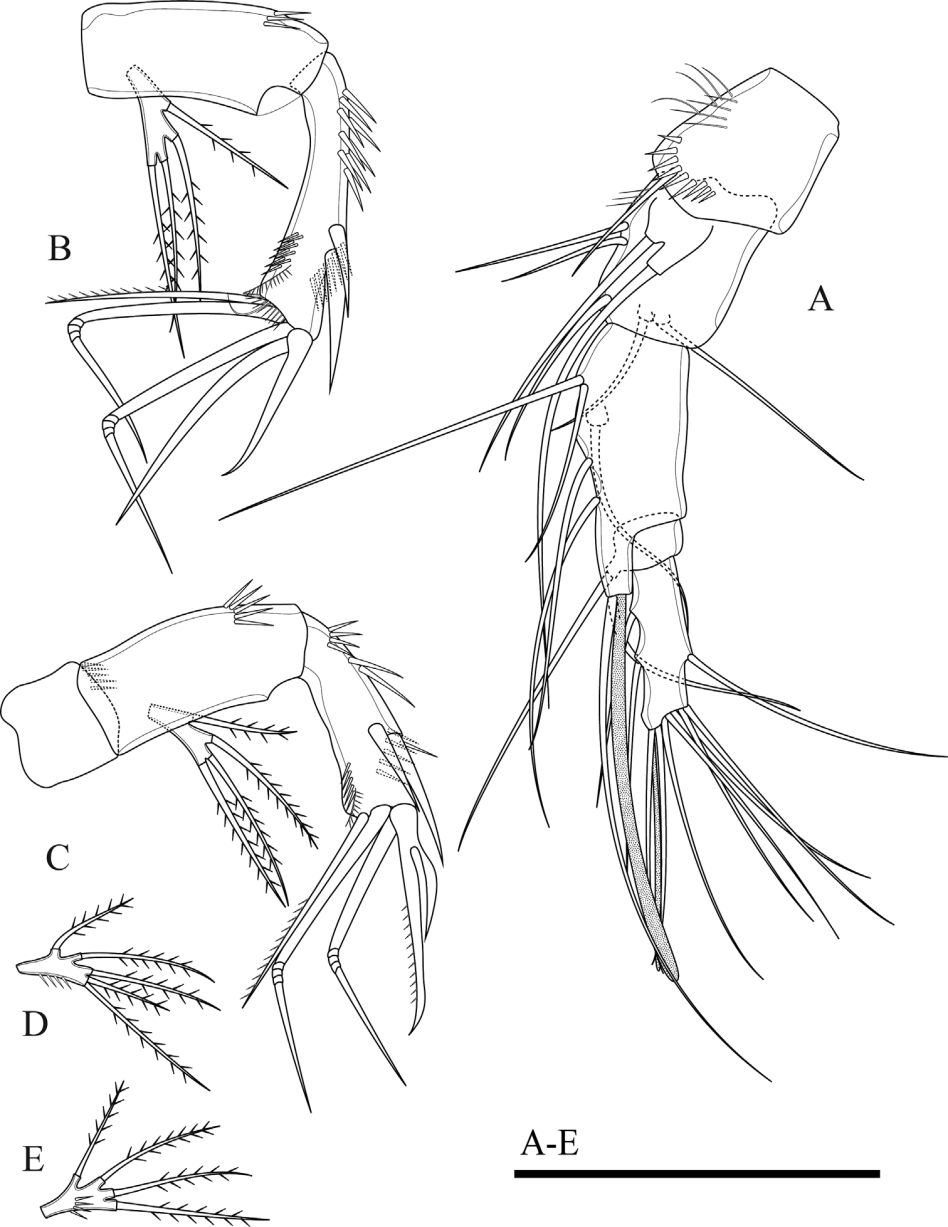
*Onychocamptustratensis* sp. n., female holotype. **A** antennule **B, C** antenna **D, E***Onychocamptustratensis* sp. n., male allotype **D, E** exopod of antenna. Scale bar: 0.05 mm.

Antennule (Fig. 10A). Short, 5-segmented, large aesthetasc on third segment and small aesthetasc on fifth segment. Surface of all segments smooth, except for medial and distal rows of small spinules on first segment. Armature formula I-[1], II-[7+(1+aesthetasc)], III-[8+aesthetasc], IV-[1], V-[9+acrothek]. Aesthetasc on third segment fuse basally to one smooth seta. Apical acrothek consists of one aesthetasc fused basally to two slender smooth setae. Only seta on first segment bipinnate, all other setae smooth.

Antenna (Figs 10B, 22A). Comprising coxa, allobasis, 1-segmented endopod, and exopod. Coxa without ornamentation. Allobasis with one row of inner spinules, with 1-segmented exopod; the latter with two apical and two lateral bipinnate setae. Free endopod with two strong sharp spines accompanied by several strong, short spinules along outer margin, distal end with five elements; one slender seta, two geniculate setae, and two strong spines.

**Figure 11.**
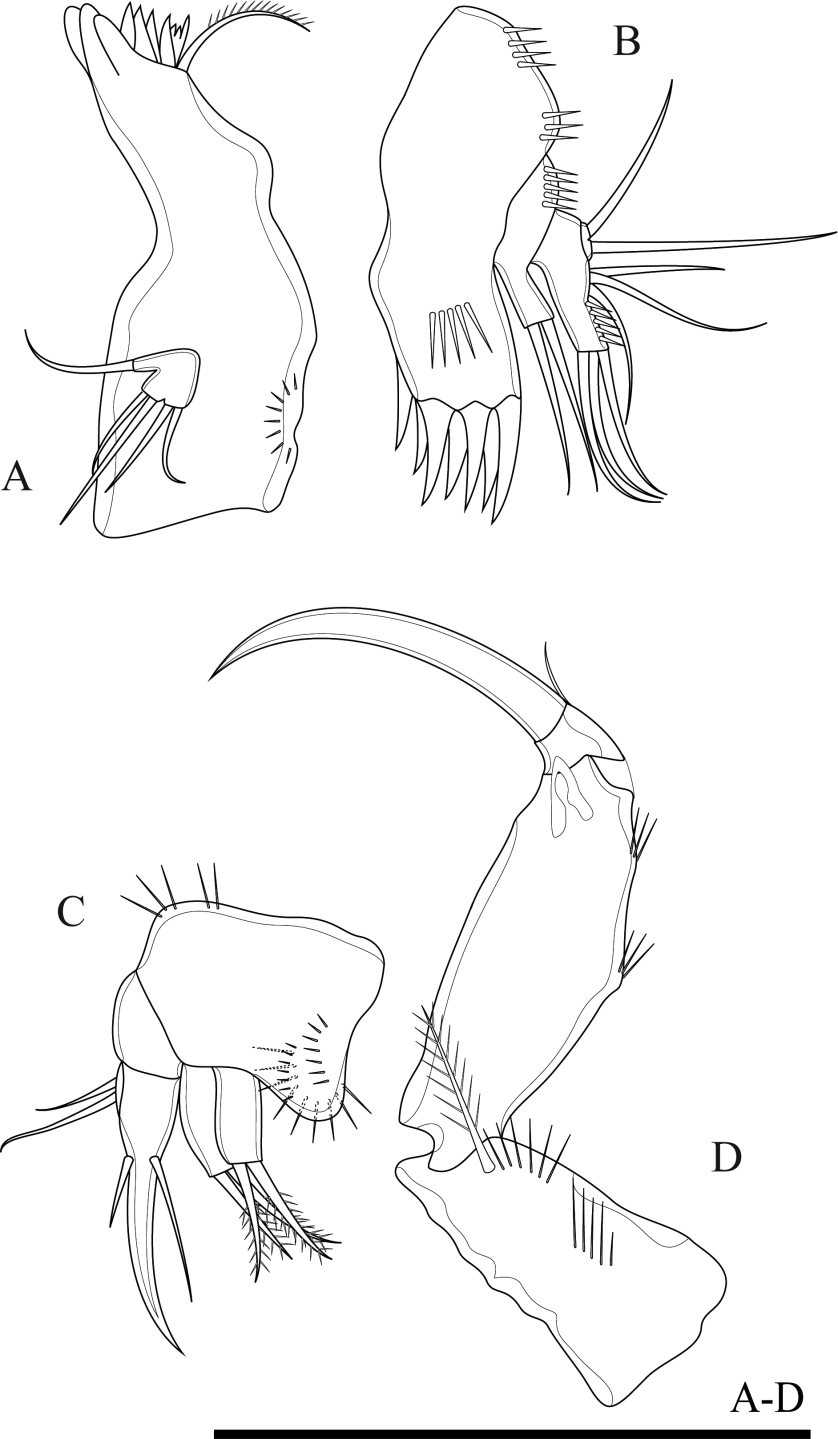
*Onychocamptustratensis* sp. n., female holotype. **A** mandible **B** maxillule **C** maxilla **D** maxilliped. Scale bar: 0.05 mm.

Mandible (Fig. 11A). Gnathobase with strong, chitinised teeth and dorsal pinnate seta along cutting edge. Mandibular palp short, 1-segmented, with five slender setae sub-equal in length.

Maxillule (Fig. 11B). Composed of robust precoxa, coxa, basis, endopod fused to basis, and 1-segmented free exopod. Precoxal arthrite with six strong apical spines, with lateral spine. Coxa with cylindrical endite bearing two smooth setae. Basis with cylindrical endite bearing three setae. Endopod incorporated to basis, with three setae. Exopod free, 1-segmented, with two sub-equal apical setae.

Maxilla (Fig. 11C). Composed of syncoxa, allobasis, and 1-segmented endopod. Syncoxa with two endites; each endite with two apical pinnate setae, outer margin with spinules. Allobasis with apical drawn out into claw, with one anterior and one posterior seta. Endopod 1-segmented, with two smooth apical setae.

Maxilliped (Fig. 11D). Subchelate, 3-segmented, comprising syncoxa, basis, and endopod. Syncoxa with one pinnate seta at outer distal corner. Basis with two transverse rows of outer spinules, one of which near base of endopod. Endopod drawn out into strong naked claw, with one small seta near base.

**Figure 12.**
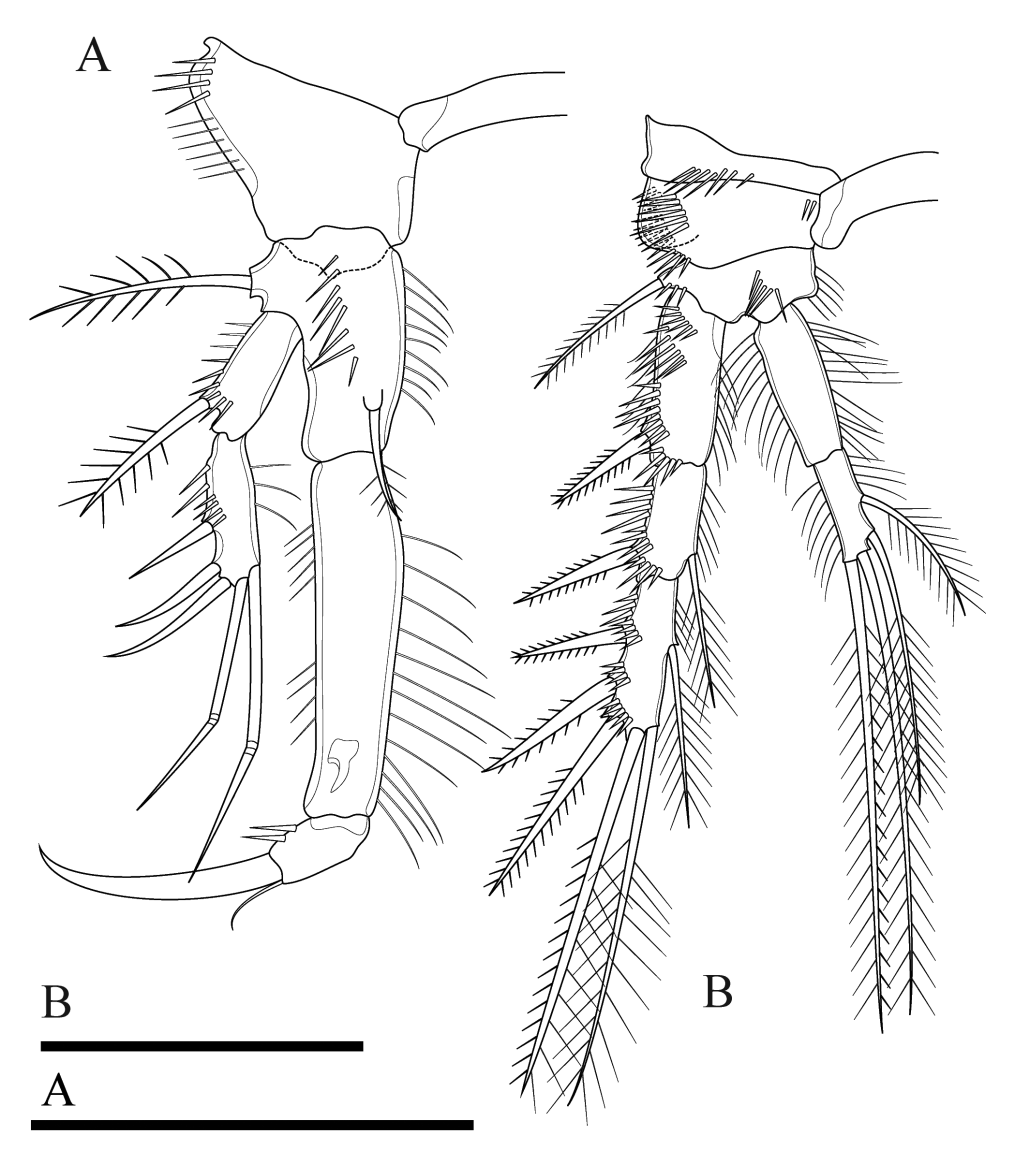
*Onychocamptustratensis* sp. n., female holotype. **A** P1 **B** P2. Scale bars: 0.05 mm.

P1 (Fig. 12A). Intercoxal sclerite naked. Precoxa small and triangular, with one row of spinules at distal margin (not shown). Coxa with one row of long outer spinules. Basis with one outer bipinnate spine and one inner plumose seta near insertion of endopod, with longitudinal row of anterior spinules medially, with long setules along inner margin. Both rami 2-segmented. Exopod reaching proximal third of enp-1; exp-1 with one bipinnate outer spine, with one row of outer spinules; exp-2 with three outer smooth spines and two apical geniculate setae, with row of outer spinules and inner setules. Enp-1 approximately 4.4 times as long as wide, with one row of outer and inner setules; enp-2 with one median strong outwardly curved claw-like smooth spine and one slender inner seta, with few outer spinules.

P2 (Fig. 12B). Intercoxal sclerite and precoxa as in P1. Coxa with two oblique parallel rows of long outer spinules (one on anterior and others on posterior surface), with one row of spinules at distal margin, and few spinules at inner margin. Basis with outer bipinnate spine, with spinules at base of spine, with one row of long spinules between exopod and endopod, and with one row of inner setules. Rami with 3-segmented exopod and 2-segmented endopod; endopod reaching tip of exp-2. Exp-1 with one outer bipinnate spine; exp-2 with one outer bipinnate spine and one inner plumose seta; exp-3 with three outer bipinnate spines, two apical elements (of which outer one spiniform seta with outer spinules and inner setules, inner element one plumose seta), and one inner plumose seta. All segments of exopod with several rows of strong outer spinules and inner setules, and only exp-1 with one row of outer setules. Enp-1 without armature, enp-2 with two apical and two inner plumose setae. All segments of endopod with one row of long outer and inner setules.

**Figure 13.**
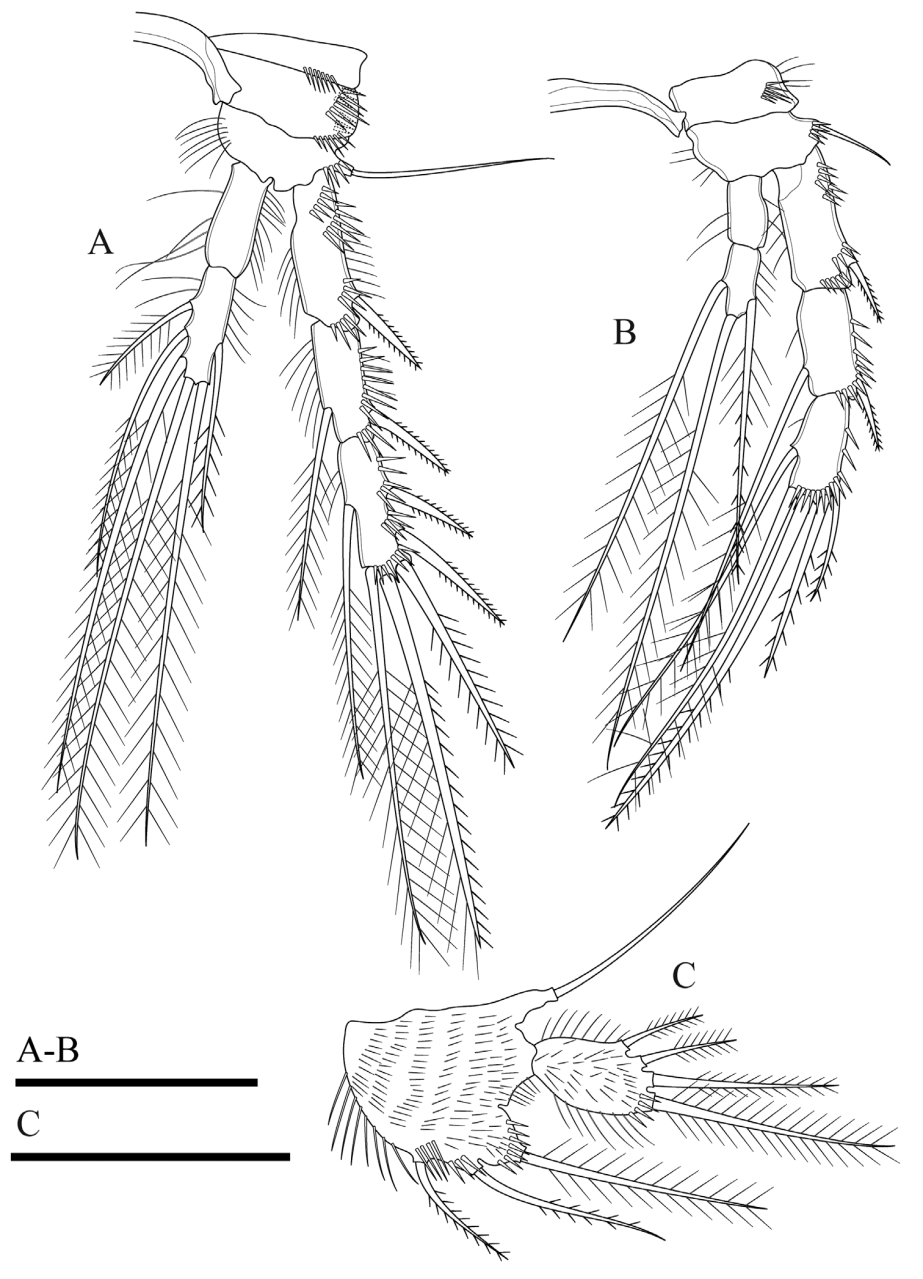
*Onychocamptustratensis* sp. n., female holotype. **A** P3 **B** P4 **C** P5. Scale bars: 0.05 mm.

P3 (Fig. 13A). Intercoxal sclerite and precoxa as in P1. Coxa with two parallel rows of long spinules along outer margin (one on anterior and other on posterior surface). Basis with one smooth outer seta; with spinules at base of seta and one row of inner setules. Segmentation of rami as in P2, endopod reaching to middle segment of exp-2. Exp-1 with one outer bipinnate spine; exp-2 with one outer bipinnate spine and one inner plumose seta; exp-3 with three outer bipinnate spines, two apical elements (of which outer one spiniform seta with outer spinules and inner setules, inner element one plumose seta), and one inner plumose seta. All segments of exopod with several rows of strong outer spinules and one row of inner setules, and exp-1 and exp-2 with one row of outer setules. Enp-1 without armature, enp-2 with one outer bipinnate seta, two apical, and three inner plumose setae. Outer and inner of all segments of endopod with long setules.

P4 (Fig. 13B). Intercoxal sclerite and precoxa (not shown) as in P1. Coxa with one row of setules and several outer spinules. Basis with one smooth outer seta; with spinules at base of seta and one row of inner setules. Rami with 3-segmented exopod and 2-segmented endopod, endopod smaller than exopod. Exp-1 with one outer bipinnate spine; exp-2 with one outer bipinnate spine and one inner plumose seta; exp-3 with two outer bipinnate spines, two apical elements (of which outer one bipinnate spine, inner element one plumose seta), and one inner plumose seta. All segments of exopod with several rows of strong outer spinules and with fewer inner setules. Enp-1 without armature, enp-2 with one outer seta with plumose proximally and bipinnate distally, one apical plumose seta, and one inner plumose seta. Outer and inner of all segments of endopod with setules.

Armature formula of P1–P4 as in Table 1.

P5 (Figs 13C, 23B). Baseoendopod and exopod separated with setules as figured. Baseoendopod with basal seta and three inner setae on endopodal lobe; one proximal bipinnate seta, one middle bipinnate seta and one apical plumose seta, with one row of spinules at base of each seta, as well as with one distal row of spinules between distal seta of baseoendopod and exopod. Exopod with four plumose setae, with row of inner and outer setules, and with spinules at base of innermost seta.

####### Description of adult male.

**Figure 14.**
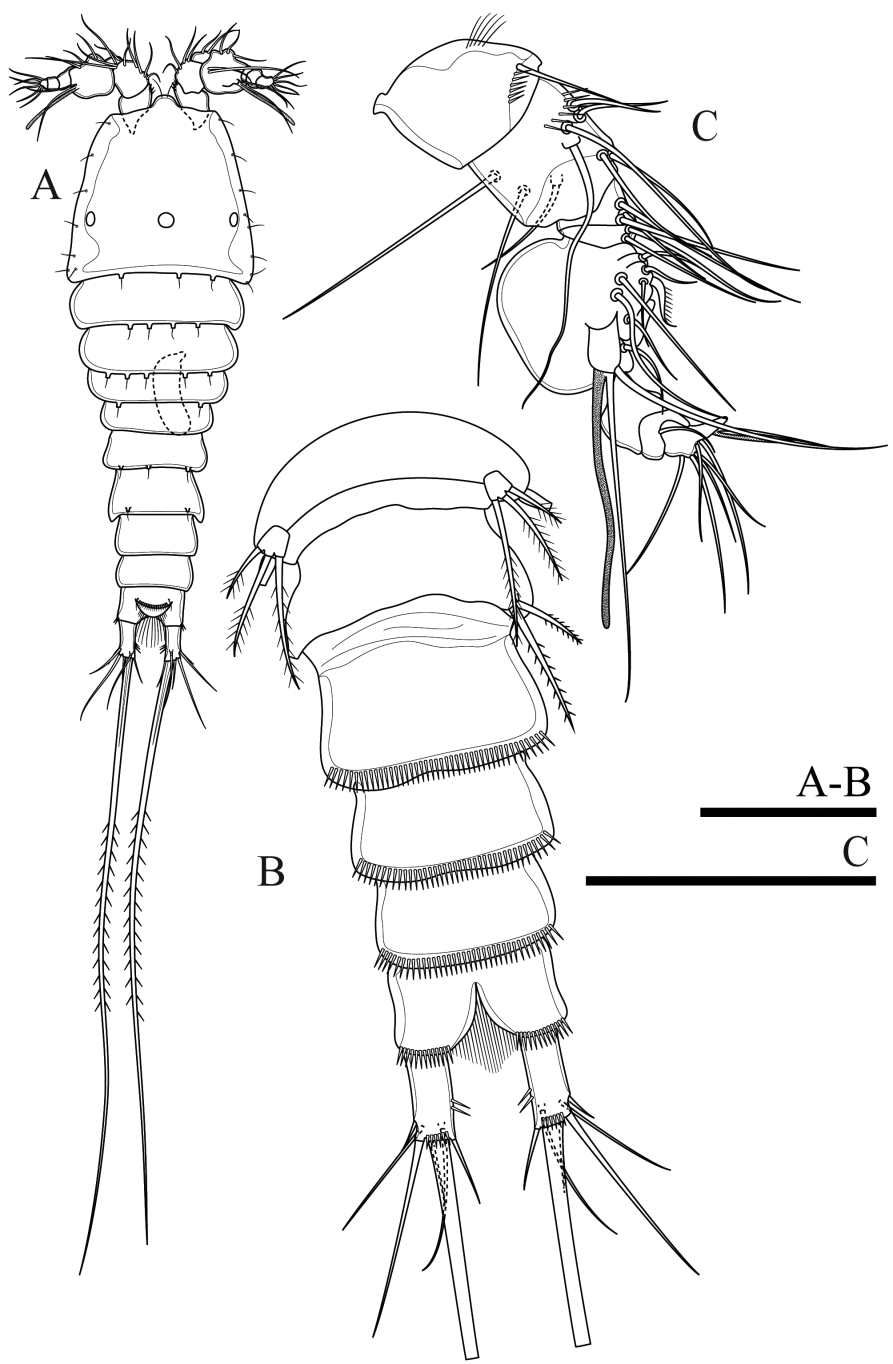
*Onychocamptustratensis* sp. n., male allotype. **A** habitus, dorsal view **B** urosome, ventral view **C** antennule. Scale bars: 0.1 mm (**A**), 0.05 mm (**B, C**).

Body (Fig. 14A, B). Total body length, measured from tip of rostrum to posterior margin of caudal rami, 340–360 µm (mean 350 µm, n = 3; 350 µm in paratype); preserved specimen colourless. Prosome approximately 1.5 times as long as urosome. Cephalothorax as long as wide, 0.5 times the length of prosome. All free thoracic somites with sensillum-bearing tubercles along posterior margin, but fifth thoracic somite (first urosomite) with additional row of posterior setules dorsally. Second and third urosomite completely separated. Second urosomite with dorsal sensillum-bearing tubercles along posterior margin. Fourth urosomite without lateral protuberances, with one posterior row of dorsal setules and ventral spinules. Ornamentation on next three urosomites as in female. Anal somite approximately 0.5 times longer than wide. Anal operculum as in female.

Caudal rami (Fig. 14A, B). As in female.

Antennule (Fig. 14C). 8-segmented, large aesthetasc on fifth segment and small aesthetasc on eighth segment. First segment with proxim al setules and subdistal outer spinules, second segment with one row of outer spinules, other segments smooth. Armature formula I-[1], II-[9], III-[3], IV-[2], V-[9+(1+aesthetasc)], VI-[0], VII-[1], VIII-[7+acrothek]. Aesthetasc on fifth segment robust. Apical acrothek consists of one aesthetasc fused basally with two slender smooth setae.

Rostrum, antenna (coxa, allobasis, and endopod), mouthparts, and P1 as in female.

**Figure 15.**
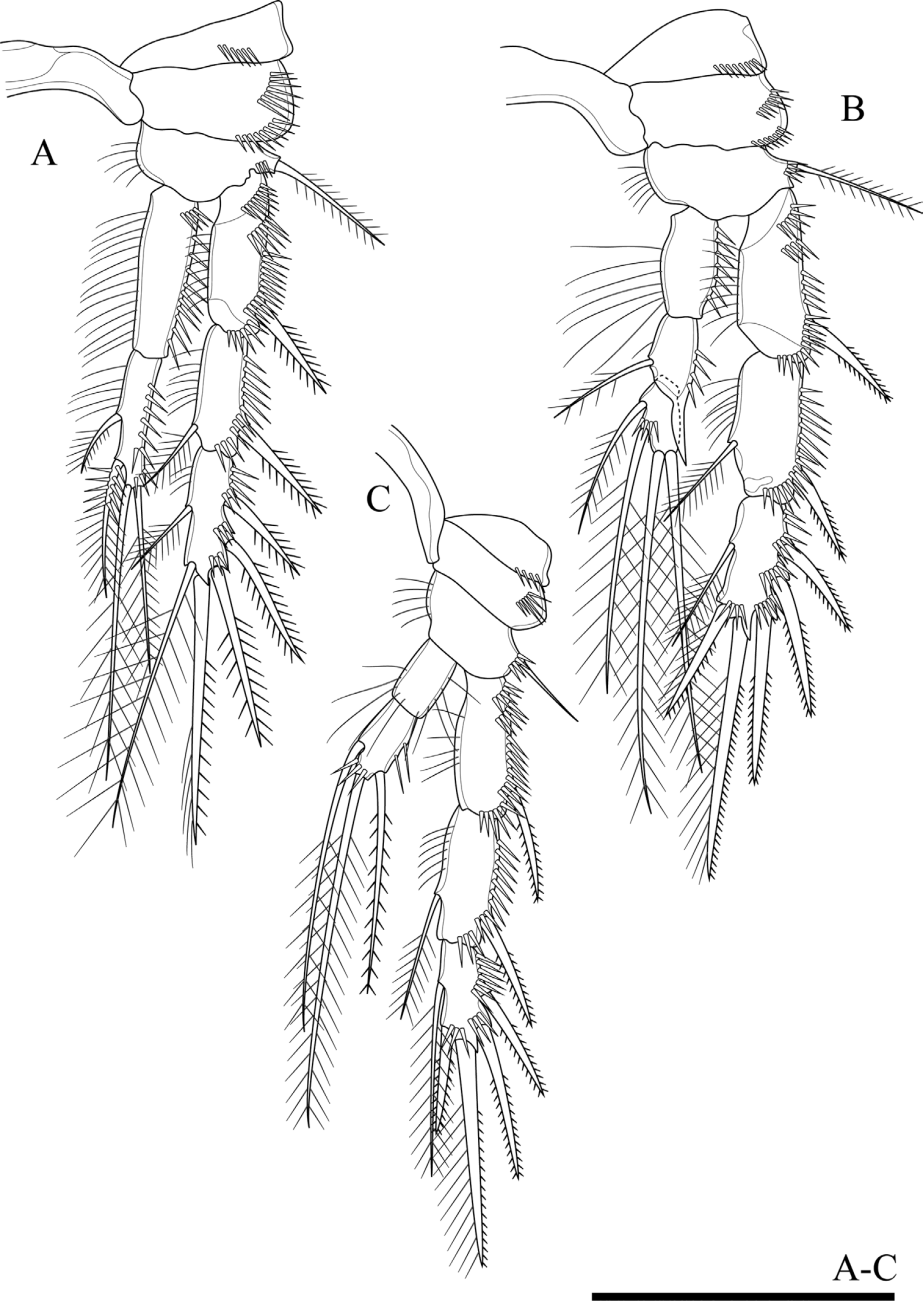
*Onychocamptustratensis* sp. n., male allotype. **A** P2 **B** P3 **C** P4. Scale bar: 0.05 mm.

P2 (Fig. 15A). Intercoxal sclerite naked. Precoxa small and triangular, with one row of spinules at distal margin. Coxa with two rows of outer spinules. Basis with one outer bipinnate seta, with spinules at base of spine, and with one row of inner setules. Rami with 3-segmented exopod and 2-segmented endopod, endopod reaching to middle of exp-3. Armature and all ornamentation of endopod as in female. Enp-1 without armature, enp-2 with two apical plumose setae and two inner plumose setae. All segments of endopod with one row of outer spinules and one row of inner setules.

P3 (Fig. 15B). Intercoxal sclerite and precoxa as in P2. Coxa with two rows of outer spinules. Basis with one outer plumose seta; with spinules at base of seta and one row of inner setules. Both rami 3-segmented, endopod reaching to middle of exp-2. Exp-1 with one outer bipinnate spine; exp-2 with one outer bipinnate spine and one inner plumose seta, inner seta much shorter than in female; exp-3 with three outer bipinnate spines, two apical elements (both spiniform seta with outer spinules and inner setules), and one inner plumose seta, inner seta much shorter than in female. All segments of exopod with several rows of strong outer spinules and one row of inner setules except exp-3. Enp-1 without armature, enp-2 with outer distal apophysis exceeding the tip of enp-3, one inner plumose seta, and with one row of outer spinules and fewer inner setules; enp-3 with two inner plumose setae and two apical plumose setae, and with one row of spinules at base of outer seta.

P4 (Fig. 15C). Intercoxal sclerite and precoxa as in P2. Coxa with one row of spinules along outer margin. Basis with one smooth seta on outer distal corner; ornamented with spinules at base of seta and one row of setules along inner margin. Rami with 3-segmented exopod and 2-segmented endopod. Exp-1 with one outer bipinnate spine; exp-2 with one outer bipinnate spine and one inner plumose seta, inner seta much shorter than in female; exp-3 with two outer bipinnate spines, two apical elements (of which outer one spiniform seta with outer spinules and inner setules, inner element one spiniform seta), and one inner plumose seta. Endopod reaching tip of exp-1; enp-1 without armature; enp-2 with one outer bipinnate seta, one apical plumose seta, and one inner plumose seta. All segments of exopod with several rows of strong spinules along outer margin, and with one row of setules along inner margin except exp-3. Armature and all ornamentation of endopod as in female, but presence of one row of spinules at base of apical seta.

Armature formula of P1–P4 as in Table 1.

P5 (Fig. 14B). With outer basal seta arising from long setophore; without endopodal lobe. Exopod with three plumose setae, outermost shortest, 2.5 times as long as segment, approximately 0.5 times as long as the middle seta.

P6 (Fig. 14B). Reduced to one minute rectangular protuberance, with one outer and one inner bipinnate seta; inner seta approximately twice as long as outer one and reaching posterior margin of next urosomite.

####### Variability.

In male, variability was observed in the exopod of antenna, four specimens with four setae and one specimen with five setae (Fig. 10D, E). In female antenna, two strong spines of distal end of endopod not fused in six specimens and fused in only one specimen (Fig. 10C).

####### Distribution.

This species is known from the type locality only. It was found in two months, January and September 2017.

###### Onychocamptus bengalensis

Taxon classificationAnimaliaHarpacticoidaLaophontidae

(Sewell, 1934)

**Figure 16.**
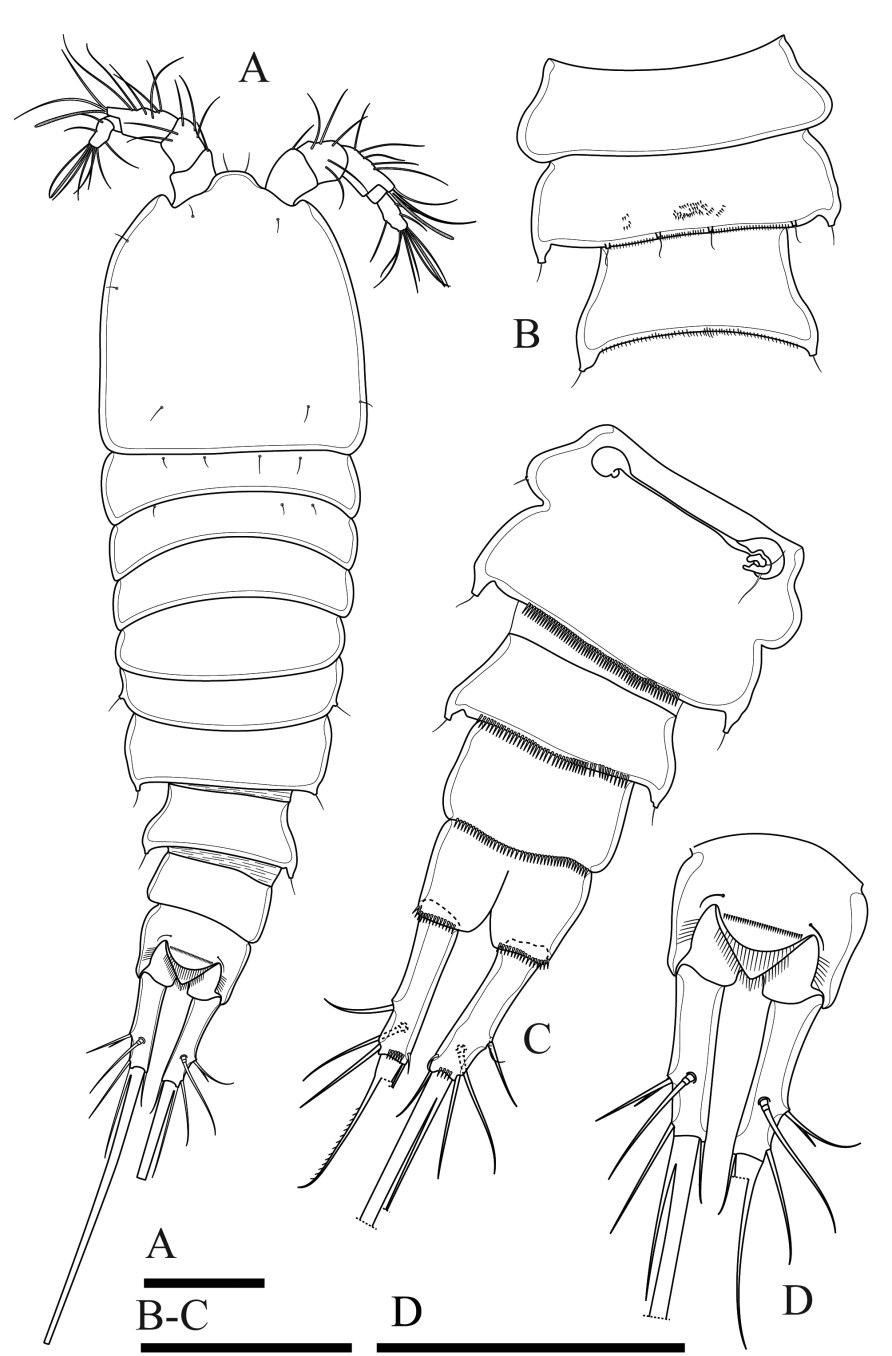
*Onychocamptusbengalensis*, female. **A** habitus, dorsal view **B** urosome, ventral view **C** genital double-somite and fourth urosomite, dorsal view **D** anal somite and caudal rami, dorsal view. Scale bars: 0.1 mm (**A**), 0.05 mm (**B–D**).

**Figure 17.**
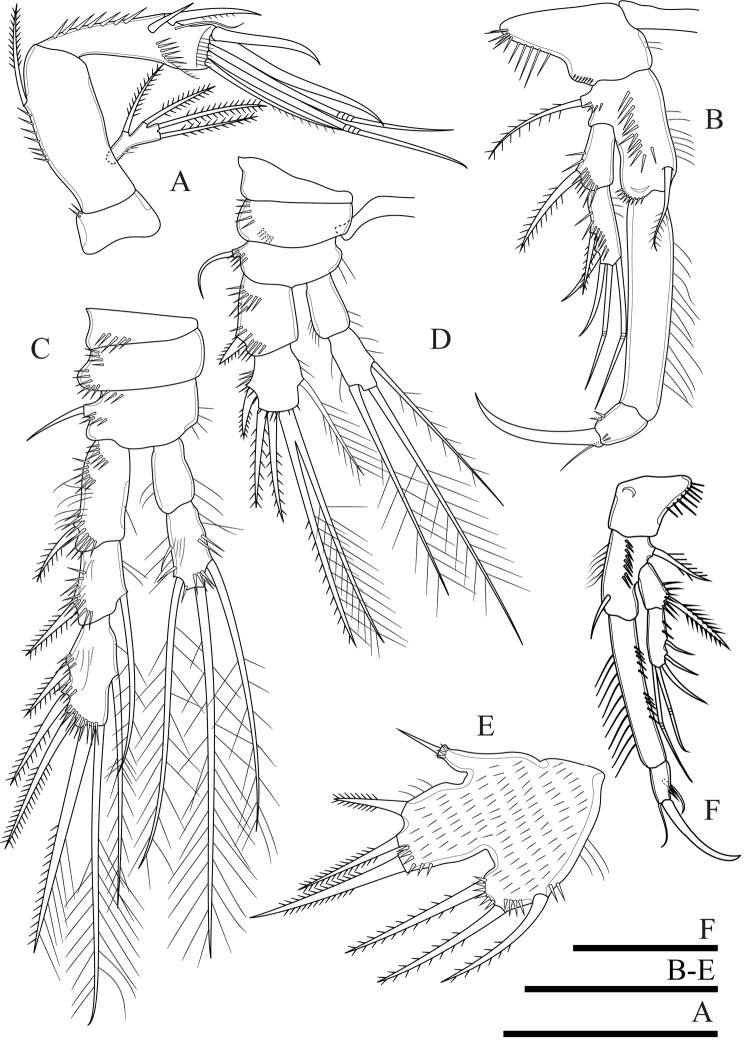
*Onychocamptusbengalensis*, female. **A** antenna **B** P1 **C, D** P4 **E** P5 **F** male P1. Scale bars: 0.05 mm.

**Figure 18.**
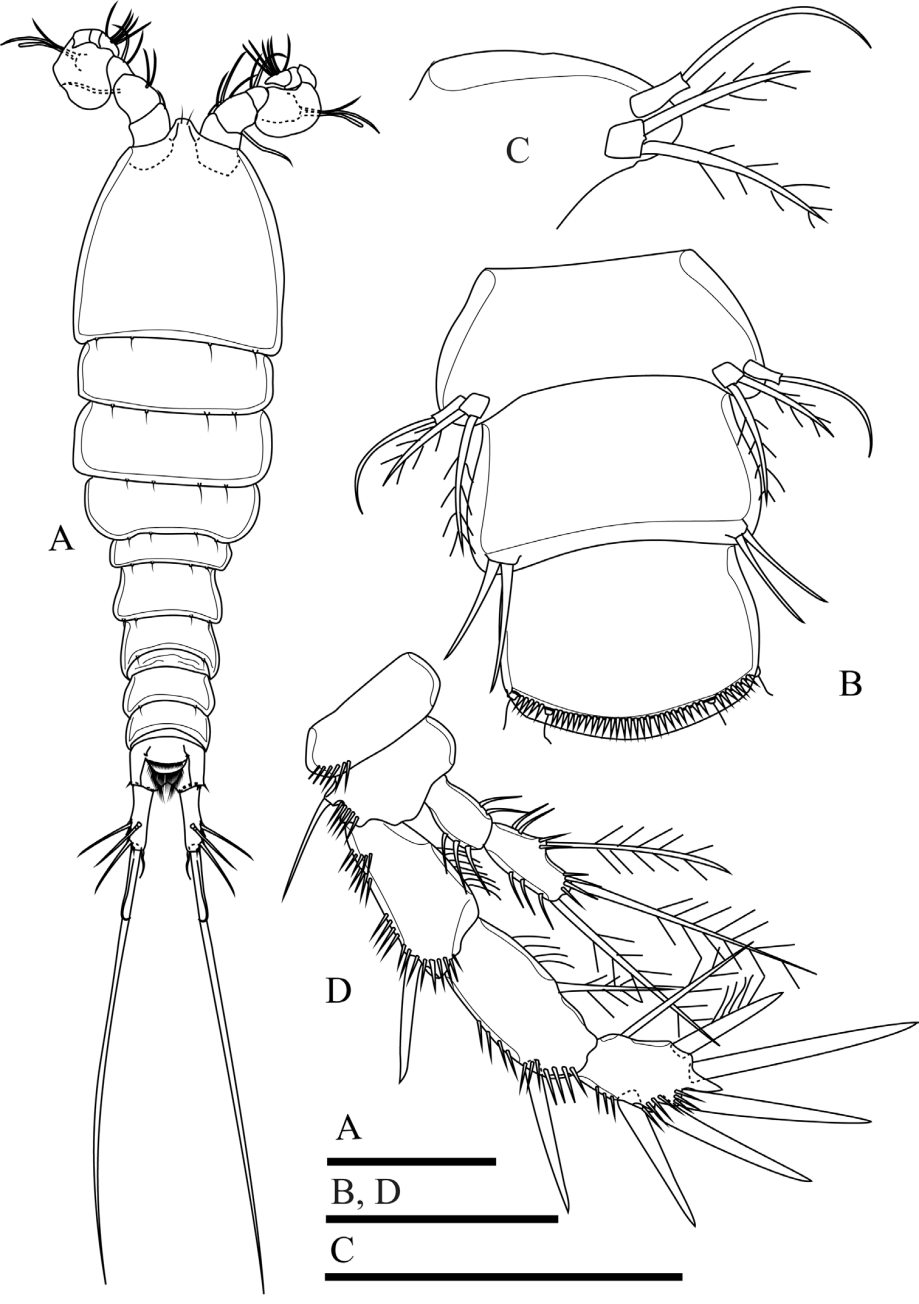
*Onychocamptusbengalensis*, male. **A** habitus, dorsal view **B** urosome, ventral view **C** left P5 **D** P4. Scale bars: 0.1 mm (**A**), 0.05 mm (**B–D**).

Figs 16 , 17 , 18 , 22B , 23C

Laophonte bengalensis Sewell, 1934: 98, fig 10a−k.
Onychocamptus bengalensis : Lang 1948: 1409, abb. 571.9, 1420, abb. 578.2; Hamond 1973: 406, figs 42−65; Song and Chang 1995: 72, 75, fig 6; Lee and Chang 2005: 40, fig. 5.

####### Material examined.

Two females and two males from Khao Thanan cave, Satun Province, southern Thailand, 07°03'43.2"N, 99°41'42.7"E; coll. C Boonyanusith and K Wongkamhaeng; 12 December 2014. One female from Samer-rach peat swamp, Trat Province, eastern Thailand, 12°28'04.0"N 102°21'20.6"E.; coll. S Maiphae and T Saetang; 25 May 2017.

####### Differential diagnosis.

Laophontidae. Caudal rami more than four times as long as wide in female and approximately three times as long as wide in male. Female P5 exopod and baseoendopod fused, endopodal lobe and exopod with three setae each. P4 exp-3 with three outer spines.

####### Redescription of adult female.

Female (Fig. 16A). Total body length measured from tip of rostrum to posterior margin of caudal rami, 530–550 µm (mean 536 µm, n = 3); preserved specimen colourless. Body covered with setules, cylindrical, gradually tapering posteriorly, with maximum width at posterior part of cephalothorax. Prosome 1.6 times as long as urosome. Rostrum small, completely fused to cephalothorax, with pair of apical sensilla. Cephalothorax as long as wide, approximately 0.5 times the length of prosome (Fig. 16A). Cephalothorax and all free thoracic somites with less developed posterior sensillum-bearing tubercles (Fig. 16A, B). Second and third urosomite fused ventrally forming genital double-somite, with dorsal and lateral remnant of original division (Fig. 16C). Posterior half of genital double-somite and subsequent somites with lateral sensillum-bearing tubercles. Other characters on urosomite as in *O.tratensis* sp. n.

Caudal rami (Fig. 16C, D). Cylindrical, parallel, 4.3 times as long as wide, with seven setae of different lengths. Anterolateral accessory seta (I) minute, inserted close to anterolateral seta (II) at distal third of rami. Posterolateral seta (III) inserted on minute protuberance. Outer terminal seta (IV) slender, fused at base to inner terminal seta (V), the latter longest, without fracture plane, approximately 0.6 times as long as body length. Inner accessory seta (VI) slender. Dorsal seta (VII) tria-rticulate, inserted at quarter of ramus. Length ratio of caudal setae to ramus length from seta I to seta VII: 0.2 : 0.5 : 0.8 : 1.1 : 9.7 : 0.3 : 0.8.

Antennule and mouthparts as in *O.satunensis* sp. n. and *O.tratensis* sp. n., except for allobasis of antenna with one bipinnate abexopodal seta (Figs 17A, 22B).

P1 (Fig. 17B), P2, P3 and P4 (Fig. 17C) as in *O.satunensis* sp. n. and *O.tratensis* sp. n., except for P4 exp-3 with three outer spines. Armature formula of P1–P4 as in Table 2.

**Table 2.** Armature formula of P1–P4 of *Onychocamptusbengalensis*, *O.vitiospinulosa*, and *O.mohammed*.

Swimming legs		Basis	Exopod	Endopod
P1	female	I-1	I-0; III,2,0	0-0; 0,I1,0
	male	I-1	I-0; III,2,0	0-0; 0,I1,0
P2	female	I-0	I-0; I-1; III,I1,1	0-0; 0,2,2
	male	I-0	I-0; I-1; III,I1,1	0-0; 0,2,2
P3	female	1-0	I-0; I-1; III,I1,1	0-0; 1,2,3
	male	1-0	I-0; I-1; III,II,1	0-0; 0-1; 0,2,2
P4	female	1-0	I-0; I-1; III,2,1	0-0; 1,1,1
	male	1-0	I-0; I-1; III,2,1	0-0; 1,1,1

P5 (Figs 17E, 23C). Baseoendopod and exopod fused; exopod rectangular. Baseoendopod with basal seta, endopodal lobe with three pinnate setae and with row of spinules at base of each seta. Exopod with three bipinnate setae, innermost longest and with spinules at its base.

P6 (Fig. 16C). Reduced to minute prominence at outer distal corner of genital field, with short slender seta.

####### Redescription of adult male.

Body (Fig. 18A). Total body length, measured from tip of rostrum to posterior margin of caudal rami, 450–470 µm (n = 2); habitus smaller than female; preserved specimen colourless. Prosome approximately 1.1 times as long as urosome. Cephalothorax as long as wide, 0.5 times as long as prosome. Other characters as in *O.satunensis* sp. n. and *O.tratensis* sp. n.

Caudal rami (Fig. 18A). slightly divergent, three times as long as wide; length ratio of caudal setae to ramus length from seta I to seta VII : 0.3 : 0.7 : 1.2 : 1.5 : 7.6 : 0.4 : 1.2.

Antennule, antenna, mouthparts, P1, P2, P3 as in *O.satunensis* sp. n. and *O.tratensis* sp. n., except for P4 exp-3 with three robust outer spines (Fig. 18D).

Armature formula of P1–P4 as in Table 2.

P5 (Fig. 18C). Baseoendopod absent. Exopod with two setae, inner approximately 1.4 times as long as outer.

P6 (Fig. 18B). Reduced to minute, rectangular protuberance with two setae apically; inner seta approximately 1.5 times as long as outer one.

####### Variability.

In male, one additional seta on left P1 enp-2 (Fig. 17F). In female, left P4 with 2-segmented, two apical elements of exp-2 fused (Fig. 17D).

*Onychocamptusbengalensis* is characterised by the relatively long caudal ramus and fusion of exopod and baseoendopod of P5 in the female. The length:width ratio of caudal ramus in female is variable (three times as long as wide in Lang (1948) and Song and Chang (1995), 3.8–4.0 times as long as wide in Lee and Chang (2005), and six times as long as wide in Hamond (1973)). The length:width ratio of the caudal ramus shown in Hamond (1973), is however, approximately 4.5 (Song and Chang 1995). The length:width ratio of the caudal ramus of the Thai specimens is 4.3. Other characters observed from the Thai specimens match well Hamond’s (1973) redescription, except for the basis of P3 and P4 with one row of setules along inner margin, and posterior margin of anal operculum with one row of spinules in the Thai material. The ornamentation of anal operculum is similar to that of the Korean specimens from the estuary of Ssangcheon Stream (Lee and Chang 2005), but it differs from the Australian specimens by lacking ornamentation at posterior margin (Hamond 1973).

####### Distribution.

This species has been recorded from Calcutta (India) (Sewell 1934), brackish lagoons in northern coastal suburbs of Sydney (Australia) (Hamond 1973), crab burrows in a mud flat a little apart from shore line in Chindo Island (Korea) (Song and Chang 1995), and from Ssangcheon Stream (Korea) (Lee and Chang 2005).

In this study, we found the species in i) Samer-rach peat swamp, Trat Province, eastern Thailand; water temperature, 27.65 °C; pH, 5.38; salinity, 6.06 ppt; conductivity, 11285.2 µS cm^-1^; total dissolved solids, 6982 mg L^-1^; dissolved oxygen, 3.86 mg L^-1^, ii) Khao Thanan cave, Satun Province, southern Thailand; this is a limestone cave which normally dries out during the dry season (April); water pH, 8.85; dissolved oxygen, 2.7 mg L^-1^; conductivity, 2.1 µS cm^-1^; salinity varies seasonally because of the effect from the sea nearby.

###### Onychocamptus vitiospinulosa

Taxon classificationAnimaliaHarpacticoidaLaophontidae

(Shen & Tai, 1963)

**Figure 19.**
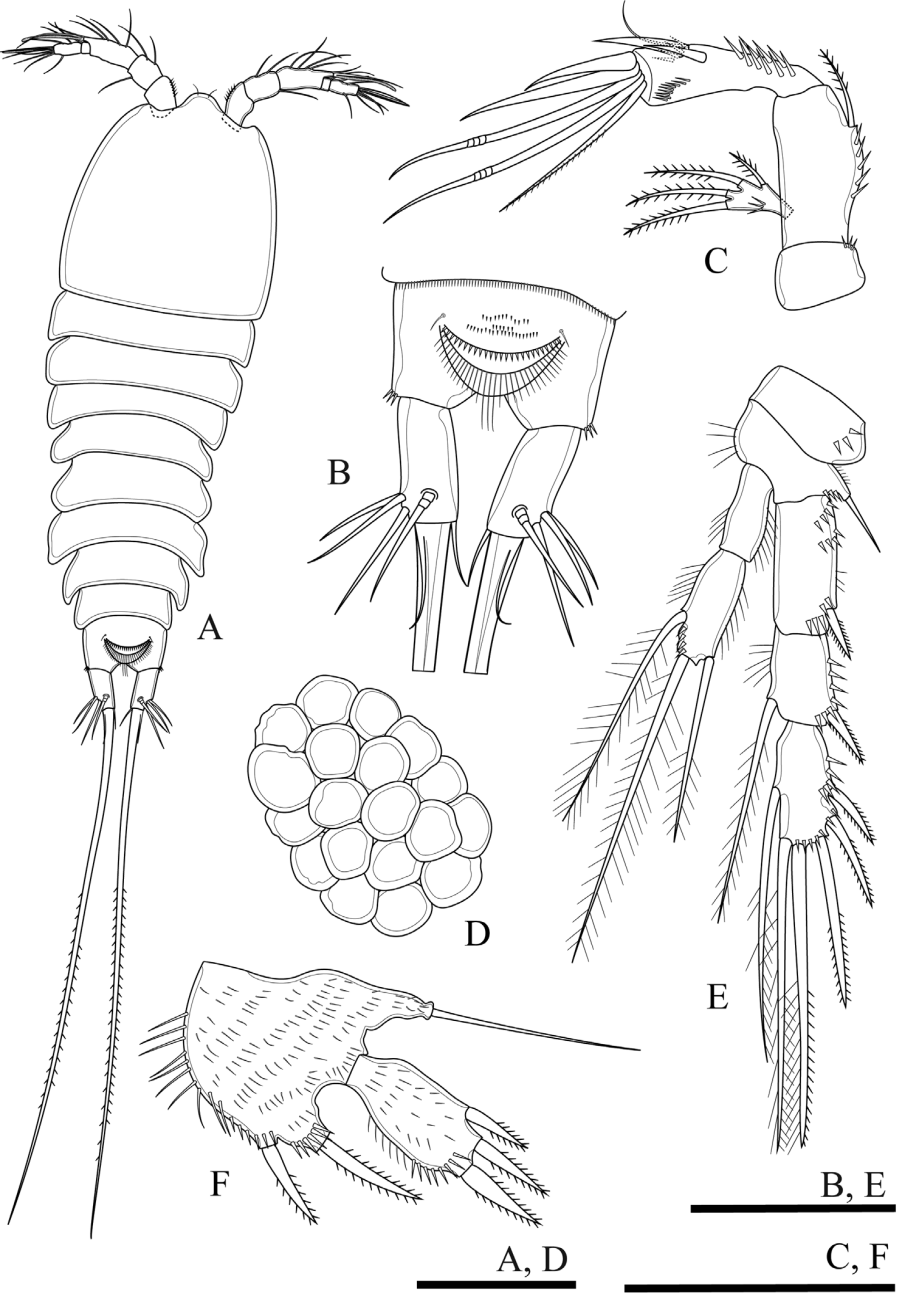
*Onychocamptusvitiospinulosa*, female. **A** habitus, dorsal view **B** anal somite and caudal rami, dorsal view **C** antenna **D** egg sac **E** P4 **F** P5. Scale bars: 0.1 mm (**A, D**), 0.05 mm (**B, C, E, F**).

**Figure 20.**
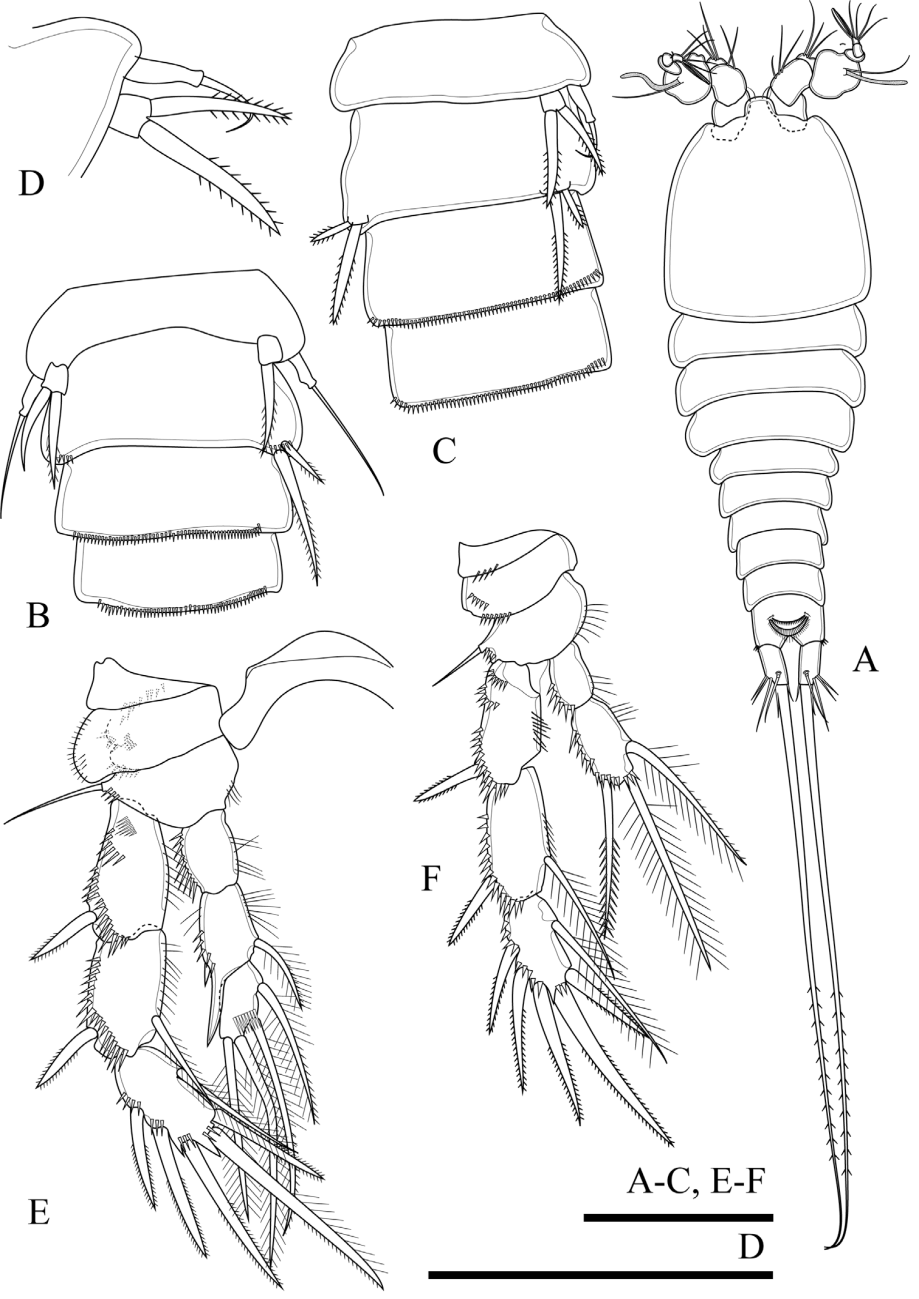
*Onychocamptusvitiospinulosa*, male. **A** habitus, dorsal view **B, C** urosome, ventral view **D** left P5 **E** P3 **F** P4. Scale bars: 0.1 mm (**A**), 0.05 mm (**B–F**).

Figs 19 , 20 , 22C , 23D

Laophonte vitiospinulosa Shen & Tai, 1963: 423, figs 42–46.
Onychocamptus mahammed vitiospinulosa : Lang 1965: 447.
Onychocamptus vitiospinulosa : Tai and Song 1979: 264, figs 147–148; Ishida 1990: 46, plate 4; Ishida and Kikuchi 2000: 34, fig 57; Lee and Chang 2005: 32, figs 1–3.

####### Material examined.

Seven females and four males from Thale-Noi Lake, Pattalung Province, southern Thailand, 07°46'30.47"N, 100°9'31.68"E; coll. S Maiphae and T Saetang; 23 October 2013.

####### Differential diagnosis.

Laophontidae. Caudal rami more than 2.3 times as long as wide in female and approximately 1.8 times as long as wide in male. P4 exp-3 with three outer spines. Female P5 exopod and baseoendopod separated, with three bipinnate spiniform setae on exopod and two on baseoendopod. Exopod of P5 male with two bipinnate spiniform setae.

####### Description of adult female.

Body (Fig. 19A). Total body length, measured from tip of rostrum to posterior margin of caudal rami, 400–460 µm (mean 423 µm, n = 7); body cylindrical, gradually tapering posteriorly. Prosome 1.5 times as long as urosome. Rostrum small, completely fused to cephalothorax, and with pair of apical sensilla. All free thoracic somites with sensillum-bearing tubercles along posterior margin. Second and third urosomite fused ventrally forming the genital double-somite, remnant of division dorsally and laterally; penultimate urosomite with row of spinules dorsally and laterally. Anal somite approximately 0.6 times as long as wide, with two rows of spinules; anal operculum poorly developed, with minute spinules along posterior border (Fig. 19B).

Caudal rami (Fig. 19B). Cylindrical, slightly convergent, 2.3 times as long as wide, with seven setae of different lengths. Position of caudal setae as in previous species. Length ratio of caudal setae to ramus length from seta I to seta VII: 0.7 : 0.9 : 1.0 : 0.7 : 11.1 : 0.4 : 1.0.

Egg sac (Fig. 19D). Ovigerous female with one rounded egg sac ventrally between pair of fifth legs, with nineteen eggs.

Antennule and mouthparts as those of previous described species, but allobasis of antenna with one bipinnate abexopodal seta (Figs 19C, 22C).

P1, P2, P3 and P4 (Fig. 19E) as those of previous species, except P4 exp-3 with three outer spines.

Armature formula of P1−P4 as in Table 2.

P5 (Figs 19F, 23D). Baseoendopod and exopod separated, covered with surface setules; baseoendopod with outer basal seta, endopodal lobe with two bipinnate spiniform setae, with one row of spinules at base of each seta; exopod with three bipinnate spiniform setae, with spinules at base of innermost seta only.

P6. Reduced to minute, rectangular protuberance, with one naked seta.

####### Description of adult male.

Body (Fig. 20A). Total body length, measured from tip of rostrum to posterior margin of caudal rami, 320–360 µm (mean 349 µm, n = 4); body cylindrical, gradually tapering posteriorly. Prosome 1.4 times as long as urosome. Rostrum small, completely fused to cephalothorax, with pair of apical sensilla. Anal somite approximately 0.8 times as long as wide, anal operculum poorly developed.

Caudal rami (Fig. 20A). As in female.

Antennule, antenna, mouthparts, P1, P2, P3 (Fig. 20E), P4 (Fig. 20F) as in *O.satunensis* sp. n. and *O.tratensis* sp. n., except P4 exp-3 with three robust, outer spines.

Armature formula of P1–P4 as in Table 2.

P5 (Fig. 20D, 23D). Baseoendopod absent. Exopod with two bipinnate spiniform setae, outer seta not reaching beyond second urosomite.

P6 (Fig. 20C). Represented by two bipinnate spiniform setae, inner approximately twice as long as outer, and inner seta reaching beyond distal margin of third urosomite.

####### Variability.

Thai specimens agree with Shen and Tai (1979), however the inner seta of the male P6 comes beyond the distal margin of four urosomite and the outer seta is approximately 1/3 as long as the inner seta in one specimen of our samples (Fig. 20B).

####### Distribution.

This species has been recorded from the delta of the Pearl River (Kwangtung Province, south China) (Shen and Tai 1963), from a stream in Okinawa and Ishigaki Island (Japan) (Ishida 1990) and from Hangetsu Lake (Shiribeshi Province, Japan) (Ishida and Kikuchi 2000), and from reed marshes of the lower reaches of Gonyangcheon Stream and Sopocheon Stream (Jindo Island, Korea) (Lee and Chang 2005).

In this study, we found this species only in Thale-Noi Lake, Pattalung Province (southern Thailand) in June, August, and October 2013. Water temperature ranged between 28.3–31.6 °C, pH of 5.22–7.83, salinity 0.1–1.2 ppt, conductivity 209.5–2385 µS cm^-1^, transparency 0.05–1.35 m, depth 0.35–1.65 m, chlorophyll a 0.27–37.53, and dissolved oxygen 2.89–5.76 mg L^-1^.

###### Onychocamptus mohammed

Taxon classificationAnimaliaHarpacticoidaLaophontidae

(Blanchard & Richard, 1891)

Fig. 21 , 22D , 23E

Laophonte mohammed Blanchard & Richard, 1891: 526, pl VI, figs 1–15; Daday 1906: Taf. 16, figs 9–16; Shen and Tai 1962: 397, figs 20–32: Tai and Song 1979: 261, figs 145–146.
Onychocamptus heteropus Daday, 1903: 157–161, figs18–24.
Laophonte calamorum Willey, 1923: 305, figs 2–4.
Onychocamptus mohammed : Lang 1948: 1417, abb. 576; Ishida and Kikuchi 2000: 34, fig 56; Lee and Chang 2005: 39–40, figs 4, 5a, b.

####### Material examined.

Four females from Ta-pom swamp, Krabi Province, southern Thailand, 08°12'50.19"N 98°46'41.24"E, coll. S Maiphae and T Saetang, on 8 December 2016.

####### Diagnosis.

Laophontidae. Caudal rami more than 2.2 times as long as wide in female. P4 exp-3 with three outer spines. Baseoendopod and exopod separated, each with three bipinnate spiniform setae.

####### Description of the adult female.

**Figure 21.**
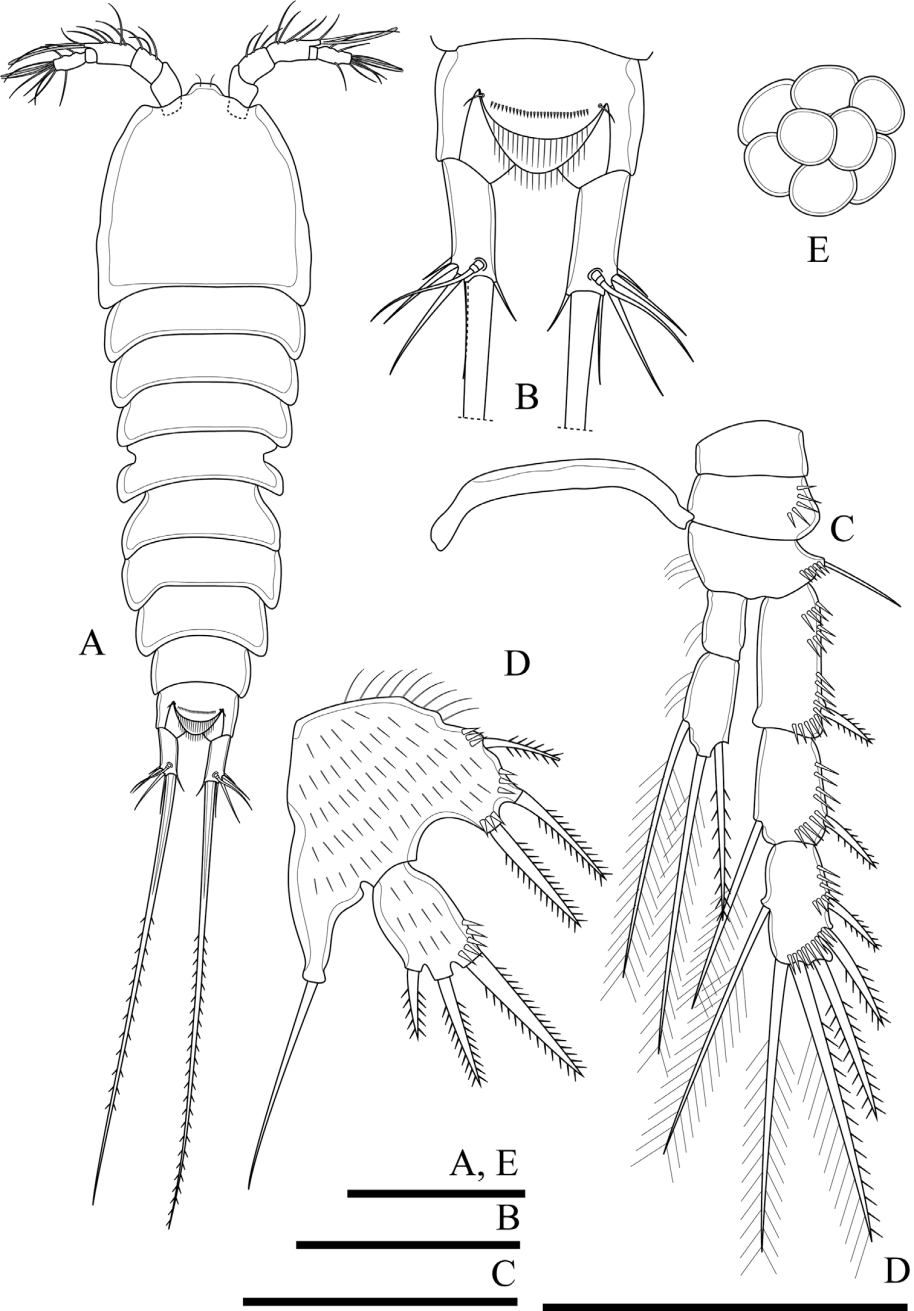
*Onychocamptusmohammed*, female. **A** habitus, dorsal view **B** anal somite and caudal rami, dorsal view **C** P4 **D** P5 **E** egg sac. Scale bars: 0.1 mm (**A, E**), 0.05 mm (**B–D**).

Body (Fig. 21A). Total body length, measured from tip of rostrum to posterior margin of caudal rami, 410–480 µm (mean 440.50 µm, n = 4); body cylindrical, gradually tapering posteriorly. Prosome 1.3 times as long as urosome. Rostrum small, completely fused to cephalothorax, with pair of apical sensilla. All free thoracic somites with sensillum-bearing tubercles along posterior margin. Second and third urosomite fused ventrally forming genital double-somite; remnant of division dorsally and laterally. Anal somite approximately 0.7 times as long as wide. Anal operculum poorly developed, with minute spinules along upper posterior border.

Caudal rami (Fig. 21B). Cylindrical, parallel, 2.2 times as long as wide, with seven setae of different lengths. Position of caudal setae as in previous species. Inner terminal seta (V) approximately 0.7 times as long as body length. Length ratio of caudal setae to ramus length, from seta I to seta VII : 0.4 : 0.6 : 0.9 : 0.8 : 8.5 : 0.4 : 1.0.

Egg sac (Fig. 21E). Ovigerous female with one oval egg sac with eight eggs ventrally between fifth pair of legs.

**Figure 22.**
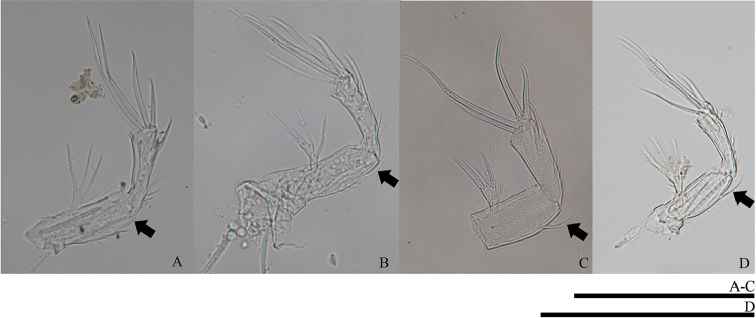
Allobasis of female antenna (digital photographs). **A***O.tratensis***B***O.bengalensis***C***O.vitiospinulosa***D***O.mohammed*. Scale bars: 0.1 mm.

Antennule and mouthparts as in previous species, but allobasis of antenna with one bipinnate abexopodal seta (Fig. 22D).

P1, P2, P3 and P4 (Fig. 21C) as in *O.satunensis* sp. n. and *O.tratensis* sp. n. except P4 exp-3 with three robust, outer spines.

Armature formula of P1−P4 as in Table 2.

**Figure 23.**
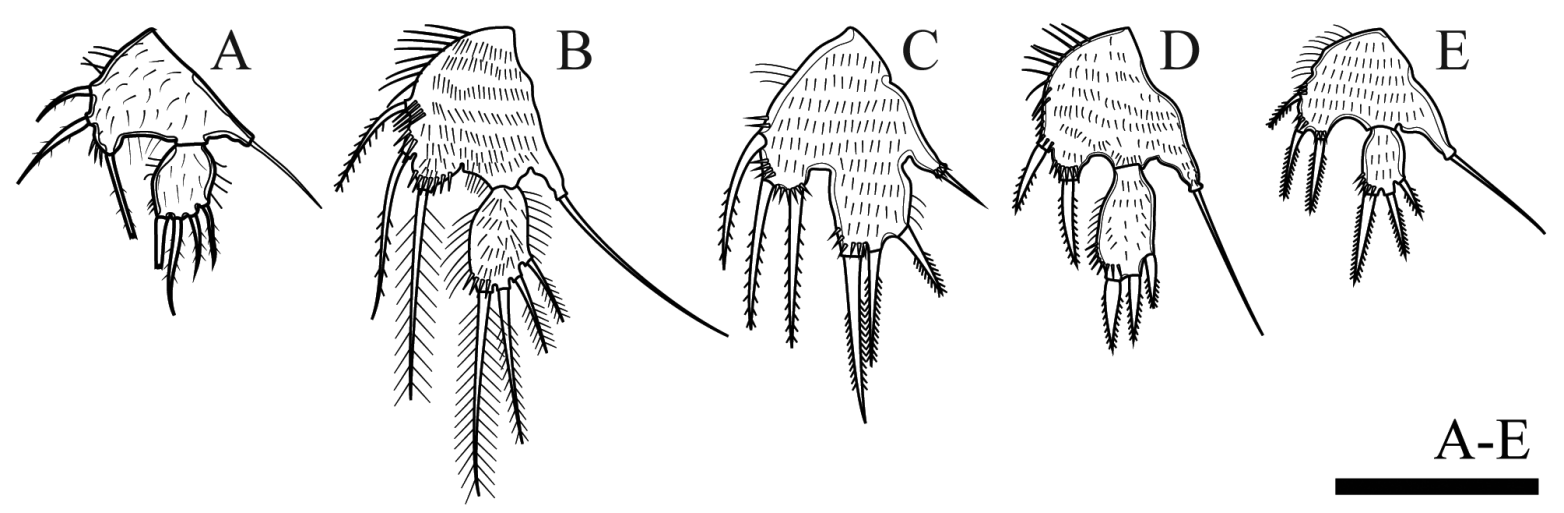
P5 of female. **A***O.satunensis***B***O.tratensis***C***O.bengalensis***D***O.vitiospinulosa***E***O.mohammed*. Scale bar: 0.05 mm.

P5 (Figs 21D, 23E). Baseoendopod and exopod separated; both rami densely covered with setules; baseoendopod with outer basal seta, endopodal lobe with three inner bipinnate spiniform setae, with one row of spinules at base of each seta; exopod with three bipinnate spiniform setae, with spinules at base of innermost seta only.

P6. Reduced to minute, rectangular protuberance, with one naked seta.

####### Variability.

The length of the baseoendopodal setae of the female P5 is variable. The original description shows the lateral most seta as the longest (Blanchard and Richard 1891). This seta is also the longest in specimens from Japan (Ishida and Kikuchi 2000). However, the middle seta is the longest in specimens from China and Korea (Shen 1962, Lee and Chang 2005), and the lateral most seta is equal to the middle seta in the Thai specimens.

####### Distribution.

This species has been found in many localities such as Wu-Li Lake (Kiangsu Province, China) (Shen and Tai 1962), in Harutori Lake (Kushiro, Japan) (Ishida and Kikuchi 2000), in Daecheong Lake and Yedang Reservior (Korea) (Lee and Chang 2005), in Vietnam (Ho and Tran 2007), and in central Thailand (Daday 1906, Watiroyram et al. 2015).

In the present study, we found the species in Ta-pom swamp (southern Thailand, Krabi Province) in December 2016, water temperature was 26.27 °C, pH 6.74, salinity 0.27 ppt, conductivity 571 µS cm^-1^, and dissolved oxygen 4.32 mg L^-1^.

###### Key to the females of *Onychocamptus* worldwide

**Table:** 

1	Exopod of P5 with four setae	**2**
–	Exopod of P5 with three setae	**4**
2	Exopod of antenna with one seta	***O.anomalus* (Ranga Reddy, 1984)**
–	Exopod of antenna with four setae	**3**
3	Cephalothorax with internal sausage-like structure	***O.satunensis* sp. n.**
–	Cephalothorax without internal sausage-like structure	***O.tratensis* sp. n.**
4	P4 exp-3 without inner seta	***O.besnardi* Jakobi, 1954**
–	P4 exp-3 with inner seta	**5**
5	Exopod of P5 fused to baseoendopod	***O.bengalensis* (Sewell, 1934)**
–	Exopod and baseoendopod of P5 completely separated	**6**
6	Baseoendopodal lobe with two setae	***O.vitiospinulosa* (Shen & Tai, 1963)**
–	Baseoendopodal lobe with three setae	**7**
7	Antenna without abexpodal seta on allobasis	***O.taifensis* Kikuchi, Daiand & Itô, 1993**
–	Antenna with abexpodal seta on allobasis	**8**
8	P4 exp-3 with two outer spines	**9**
–	P4 exp-3 with three outer spines	***O.mohammed* (Blanchard & Richard, 1891)**
9	Caudal rami 2.8 times as long as wide, genital field with rows of spinules near P6	***O.krusensterni* Schizas & Shirley, 1994**
–	Caudal rami twice as long as wide, genital field without rows of spinules near P6	***O.fratrisaustralis* Gómez, 2001**

###### Key to the males of *Onychocamptus* (the male of *O.fratrisaustralis* Gómez, 2001 remains unknown and has not been included here) worldwide

**Table:** 

1	Exopod of P5 with three setae	**2**
–	Exopod of P5 with two setae	**4**
2	Exopod of antenna with one seta	***O.anomalus* (Ranga Reddy, 1984)**
–	Exopod of antenna with four setae	**3**
3	Cephalothorax with internal sausage-like structure	***O.satunensis* sp. n.**
–	Cephalothorax without internal sausage-like structure	***O.tratensis* sp. n.**
4	P4 exp-3 without inner seta	***O.besnardi* Jakobi, 1954**
–	P4 exp-3 with inner seta	**5**
5	Caudal rami more than four times as long as wide	***O.bengalensis* (Sewell, 1934)**
–	Caudal rami less than or approximately three times as long as wide	**6**
6	Antenna without abexpodal seta on allobasis	***O.taifensis* Kikuchi, Daiand & Itô, 1993**
–	Antenna with abexpodal seta on allobasis	**7**
7	P4 exp-3 with two outer spines	***O.krusensterni* Schizas & Shirley, 1994**
–	P4 exp-3 with three outer spines	**8**
8	Inner seta on P6 approximately or less than twice as long as outer seta	***O.mohammed* (Blanchard & Richard, 1891)**
–	Inner seta on P6 approximately or more than 2.5 times as long as outer seta	***O.vitiospinulosa* (Shen & Tai, 1963)**

## Discussion

The two new species identified in this study can confidently be assigned to the genus *Onychocamptus* based on the combination of characteristics mentioned by Huys and Lee (2000) and Lee and Huys (1999): (1) female antennule with five segments, (2) male antennule with up to three segments distal to the geniculation, (3) caudal ramus with strongly developed seta V, (4) mandibular palp uniramous, (5) maxilliped with one seta on the syncoxa, (6) P1 with two-segmented exopod, (7) enp-1 of P1 without inner seta, (8) endopodal lobe of P5 with three setae, (9) inner distal element of P3 and P4 exp-3 showing sexual dimorphism (setiform in females, but spiniform in males) and (10) male P3 enp-2 with one inner seta. Based on the retention of the ancestral inner seta on P3 enp-2 of the male and geographical distribution, Lee and Huys (1999) suggested that *Onychocamptus* belongs to an ancient lineage which probably diverged from the stem group of the family Laophontidae.

When compared to the representatives of *Onychocamptus*, the two new species share the highest similarity with the Indian species *O.anomalus*, and the following features were common to all species: absence of the abexopodal seta on antenna, P4 exp-3 with only two outer spines, and four and three setae on the exopod of P5 of the female and the male respectively (Table 3). This suggests a close phylogenetic relationship among Thai and Indian species, as these three species are different from all other members of the genus with regard to several characteristics. For example, *O.taifensis* lacks the abexopodal seta of antenna (Kikuchi et al. 1993), the outer spine on P4 exp-3 is reduced in *O.krusensterni* (Schizas and Shirley 1994), and both *O.taifensis* and *O.krusensterni* have three and two setae on the P5 exopod of the female and the male, respectively. A detailed comparison of the characteristics and geographical distribution of the ten species is provided in Table 3. Three groups which comprise the most closely related species are evident: the American species group (*O.fratrisaustralis*, *O.krusensterni*, and *O.besnardi*), the South Asian species group (*O.anomalus*, *O.satunensis* sp. n, and *O.tratensis* sp. n.), and the group containing the remaining species. The American species group is characterised by reduction of the spine on P4 exp-3 and the presence of the abexopodal seta. The South Asian species group is characterized by the presence of one additional seta on the P5 exopod and the absence of one abexopodal seta. The remaining species show retention of three spines on P4 exp-3.

With the description of the two new species and new records of the two species from Thailand, the number of *Onychocamptus* species recorded in Thailand has now increased from one to five. Sampling of cave-dwelling copepods in this country has revealed a large number of new species of the genera *Elaphoidella, Bryocyclops, Fierscyclops*, and *Thermocyclops* (Boonyanusith et al. 2013, Brancelj et al. 2010, Karanovic et al. 2017, Watiroyram 2018, Watiroyram and Brancelj 2016, Watiroyram et al. 2012, 2015a, b), and a new genus, *Siamcyclops*, from west Thailand (Boonyanusith et al. 2018). Most of the samples were collected from a single cave.

Based on previous studies on cave-dwelling copepods in more than twenty caves in other regions of the country (Boonyanusith 2013; Watiroyram 2012) and nine caves in Satun and Songkhla province by the first and the third authors, it seems that *O.satunensis* sp. n. was encountered only in its type locality. The occurrence of *O.satunensis* in caves is interesting, as all the other species were only recorded from surface water habitats near the coast of all continents (Table 3). Morphological comparison of all swimming legs clearly showed a low degree of differentiation. This finding indicates the relatively recent speciation of this representative of the genus. Watiroyram et al. (2017) suggested that penetration into groundwater during the Quaternary glaciation might have resulted in the speciation of several cave-dwelling copepods in this country. This might explain why a lesser number of species have been found in Holarctic countries. This may also be the reason why the distribution of several *Onychocamptus* species is, in general, fragmentary, most species are found in a narrow distribution range (Table 3): *O.fratrisaustralis* and *O.krusensterni* from North America, *O.besnardi* from Central and South America, *O.anomalus* from South Asia, *O.satunensis* sp. n. and *O.tratensis* sp. n. from Southeast Asia, and *O.taifensis* and *O.vitiospinulosa* from East Asia along the Western coast of the North Pacific Ocean. In our case, *O.satunensis* sp. n. is very morphologically similar to *O.tratensis* sp. n., and this is indicative of their very close phylogenetic relationship. We assumed that a *O.tratensis*-like common ancestor was distributed in both ancient East and South of Thailand during the connection of the East and South of Thailand by landmass at 120 m above sea level in the late Pleistocene (Voris 2000), and that a population of common ancestors might have penetrated the caves before the rising of the sea level up to 5 m above the previous sea level in the Miocene separated them from each other.

## Supplementary Material

XML Treatment for Onychocamptus satunensis

XML Treatment for Onychocamptus tratensis

XML Treatment for Onychocamptus bengalensis

XML Treatment for Onychocamptus vitiospinulosa

XML Treatment for Onychocamptus mohammed
